# De novo design of obligate ABC-type heterotrimeric proteins

**DOI:** 10.1038/s41594-022-00879-4

**Published:** 2022-12-15

**Authors:** Sherry Bermeo, Andrew Favor, Ya-Ting Chang, Andrew Norris, Scott E. Boyken, Yang Hsia, Hugh K. Haddox, Chunfu Xu, T. J. Brunette, Vicki H. Wysocki, Gira Bhabha, Damian C. Ekiert, David Baker

**Affiliations:** 1 Department of Biochemistry, University of Washington, Seattle, WA USA; 2 Institute for Protein Design, University of Washington, Seattle, WA USA; 3 Biological Physics, Structure and Design Graduate Program, University of Washington, Seattle, WA USA; 4 Molecular Engineering and Sciences Institute, University of Washington, Seattle, WA USA; 5 Department of Cell Biology, New York University School of Medicine, New York, NY USA; 6 Department of Chemistry and Biochemistry, The Ohio State University, Columbus, OH USA; 7 Resource for Native Mass Spectrometry Guided Structural Biology, The Ohio State University, Columbus, OH USA; 8 Howard Hughes Medical Institute, University of Washington, Seattle, WA USA; 9 Department of Microbiology, New York University School of Medicine, New York, NY USA

**Keywords:** Protein folding, Protein folding

## Abstract

The de novo design of three protein chains that associate to form a heterotrimer (but not any of the possible two-chain heterodimers) and that can drive the assembly of higher-order branching structures is an important challenge for protein design. We designed helical heterotrimers with specificity conferred by buried hydrogen bond networks and large aromatic residues to enhance shape complementary packing. We obtained ten designs for which all three chains cooperatively assembled into heterotrimers with few or no other species present. Crystal structures of a helical bundle heterotrimer and extended versions, with helical repeat proteins fused to individual subunits, showed all three chains assembling in the designed orientation. We used these heterotrimers as building blocks to construct larger cyclic oligomers, which were structurally validated by electron microscopy. Our three-way junction designs provide new routes to complex protein nanostructures and enable the scaffolding of three distinct ligands for modulation of cell signaling.

## Abstract (web summary)

ABC-type heterotrimeric proteins can be designed de novo using coiled coils and helical bundles as starting scaffolds, extended using helical repeat proteins and then used as building blocks for higher-order oligomeric assemblies.

## Main

**Extended Data Fig. 1:**
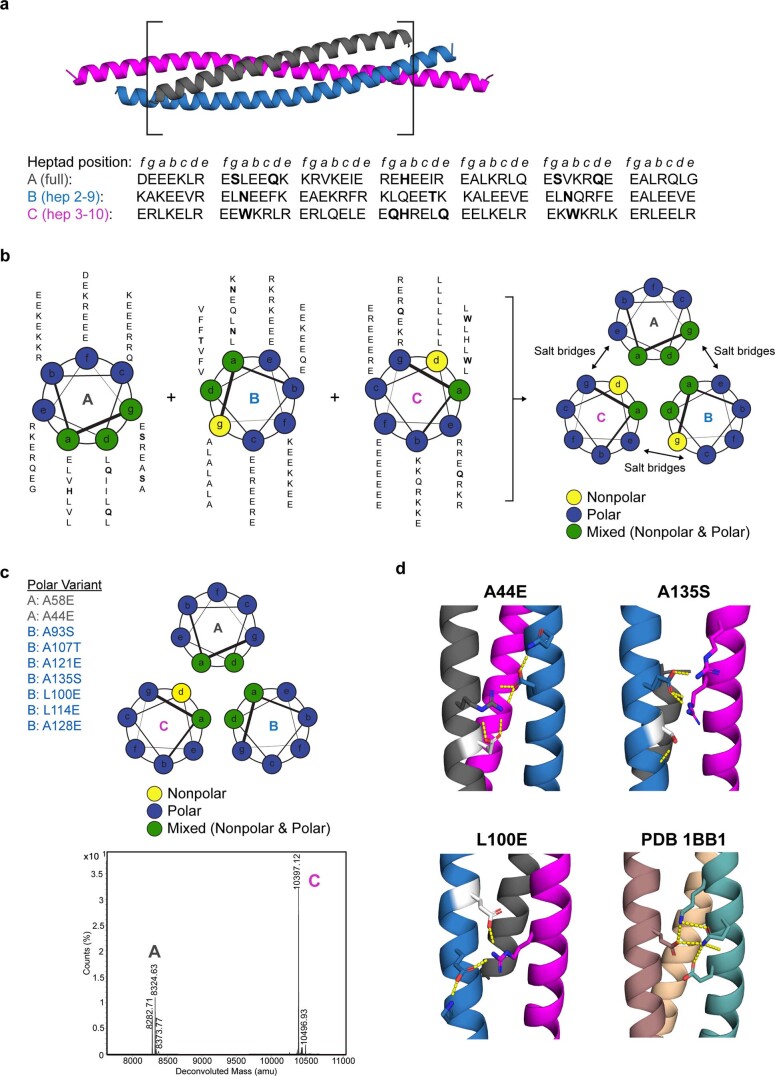
Helical wheel for coiled coil DHT01 and mutations at position g affect ABC assembly. **a** Shared interfaces of all three chains of the heterotrimer are broken up by heptads and helical wheel positions, with the amino acid letters in bold indicating the residues found by Monte Carlo HBNet. Residues contributing to the designed hydrogen bond networks are found at a, d, e, and g positions. **b** A helical wheel was drawn for each chain and then combined to form a heterotrimer wheel, with positions color-coded to indicate the presence of nonpolar, polar, or mixed (nonpolar and polar) residues. Letters in bold again match the residues found by Monte Carlo HBNet. **c** A helical wheel for a heterotrimer variant that now has only polar residues at position g for chains A and B, with respective mutations colored by chain (gray for A, blue for B). Experimental validation of this design by LC-MS indicates the absence of chain B in the IMAC pull-down eluate. **d** Three mutations of the variant heterotrimer from **c** are shown in cartoon and stick representation, with the mutated amino acid colored in white. It was hypothesized that polar or charged amino acids could help contribute to ionic interactions, which were found to be important for mediating specificity in PDB 1BB1, a previously designed ABC coiled coil peptide^3^. One example of an ionic interaction designed for this peptide is shown to the lower right, as determined by a 1.8 Å x-ray crystal structure.

Over half of the proteins found in the Protein Data Bank (PDB) assemble to form homo-oligomers or hetero-oligomers^1^. The most abundant hetero-oligomers in nature are heterodimers; heterotrimers are less widespread with notable examples including the G-proteins, the synaptic SNARE complex, collagen and laminin. Heterotrimers can either be composed of two distinct chains (AAB type) or three distinct chains (ABC type, which we refer to as simply ABC throughout the text). There has been some success with designing ABC heterotrimers using coiled coils and collagen triple helices as starting scaffolds. Early coiled coil work used preferential electrostatic interactions between ion pairs at solvent-exposed e and g positions^2–4^ (in the standard helical wheel representation; see Extended Data Fig. 1 for an example) to engineer heterotrimer specificity; similar approaches have been used for coiled coil heterodimers^5^. Collagen mimetic peptide design has also utilized complementary pairing of surface electrostatic interactions to promote heterotrimer assembly^6–8^ and destabilize competing states^9–12^. ABC heterotrimers have also been designed using steric matching approaches^13–16^ and metal template-mediated strategies^17,18^. In all of these previously designed ABC systems, the individual chains were relatively short peptides that were synthesized using peptide chemistry. However, to be useful for the design of larger multichain protein assemblies, the components must be producible in cells and the heterotrimeric interfaces must be sufficiently robust to drive assembly of the larger system.

We set out to design cooperatively assembling protein heterotrimers in which only the ABC species forms. This is a more difficult challenge than designing heterodimers because of the larger number of alternative structures: for a two-chain system, there are only four alternative species (A, B, AA and BB), while a three-chain system has 15 alternative species (A, B, C, AB, AC, BC, AAA, BBB, CCC, AAB, ABB, AAC, ACC, BBC and BCC). We reasoned that cooperatively assembling ABC heterotrimers could be designed by burying polar residues capable of making hydrogen bond networks in the core and by incorporating large aromatic residues for implicit negative design^19^ against non-ABC assemblies—such sidechains can complicate core packing in undesirable alternative states by causing steric clashes or large cavities.

## Results

### Design and characterization of ABC heterotrimer coiled coils

**Fig. 1:**
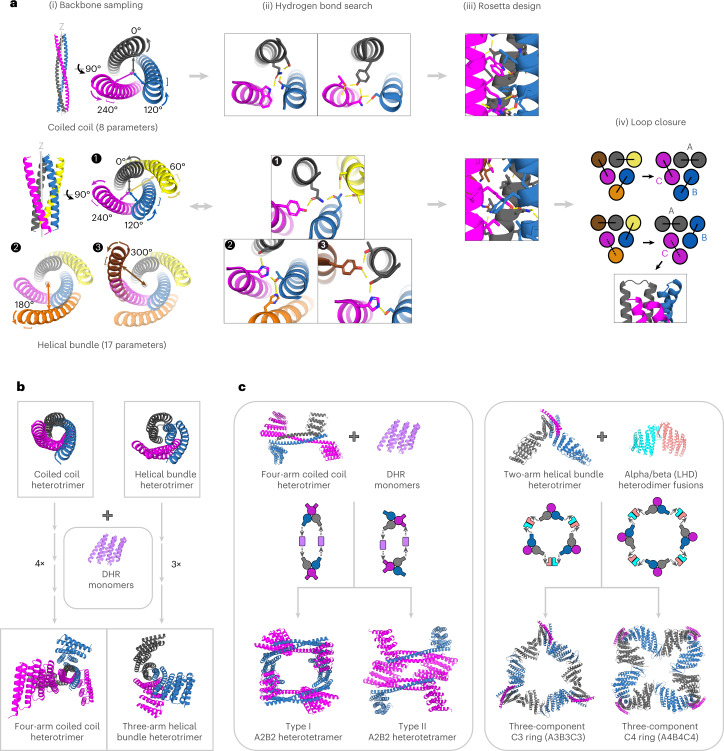
Overview of the design approach. **a**, Scaffold sampling for base heterotrimer design. (i) To generate ABC coiled coils, the three helical parameters (radius (*R*; double-headed arrows), rotation around the helical axis (Δɸ; curved arrows) and relative displacement along the *z* axis (*Z*_off_; square brackets)) are independently sampled for each of the three helices (eight parameters in total, as the *Z* offset for the first helix is zero). (ii) These backbones are then coupled to Monte Carlo HBNet to find hydrogen bond networks spanning all three helices. For the helical bundle approach, to break down the combinatorial explosion that arises when all 6 × 3 − 1 = 17 helical parameters are sampled simultaneously, a stepwise approach is taken to divide the search problem into three steps. (1) Backbone sampling is carried out for the three inner helices and one outer helix and Monte Carlo HBNet is used to identify the subset of backbones that can host a network spanning all four helices. (2) For this subset, a fifth helix is then sampled and Monte Carlo HBNet is used to identify backbones with networks that span this helix and the three central helices. (3) To these selected backbones, a sixth helix is added and a final Monte Carlo HBNet search is carried out to identify networks involving this new helix and the three inner helices. (iii) Backbones from both approaches can be optionally trimmed, packed with phenylalanine and other aliphatic residues in the core and decorated with charged residues at the surface to enhance electrostatic interaction across the chains. (iv) To the six-helix bundles, short designed connecting loops are added to generate three helical hairpin units. Two possible loop combinations are shown, with clockwise or counterclockwise closure and with loops all facing the same direction (solid lines) or loops at opposing terminal ends (dashed lines). **b**, Designed helical repeat (DHR) monomers can be rigidly joined to both coiled coil and helical bundle heterotrimers through single fusions, which can then be combined to make four- and three-arm heterotrimers, respectively. **c**, Heterotrimer arms can be combined with other designed building blocks to create higher-order nanostructures, such as A2B2 heterotetramers or A3B3C3/A4B4C4 hetero-oligomers.

The simplest case of an ABC heterotrimer is a coiled coil, in which each chain is a single helix (Fig. 1a). A generalized Crick coiled coil parameterization^20,21^ approach was used to sample the helical phase (Δɸ_1_), supercoil radius (*R*) and *Z* offset (*Z*_off_) for each helix individually. The supercoil phases (Δɸ_0_) were restricted to 0°, 120° and 240°, while the supercoil (ω_0_) and helical twist (ω_1_) were kept at ideal values (−2.85 and 102.85, respectively) to generate left-handed supercoils with 3.5 residues per turn and a seven-residue (heptad) periodicity across two turns. These poly-alanine backbones were then input to Rosetta Monte Carlo HBNet^22–24^, which places residues with polar groups across the interface such that all heavy atom donors and acceptors form hydrogen bond networks. We searched for backbones capable of hosting three hydrogen bond networks simultaneously, with each network spanning all three helices and at least two networks contributing one tyrosine or tryptophan residue each. The helices for chains B and A were then trimmed by two and four heptads, respectively, to make it easier to keep track of each chain during downstream characterization and to allow for additional electrostatic interactions between the termini. RosettaDesign^25^ was then used to optimize the amino acid sequence of the remaining residues, keeping the identities and conformations of the HBNet residues fixed (we hypothesized that fully hydrophobic heptads above and below the networks would help to keep the hydrogen-bonding residues in place). Designs were filtered by hydrogen bond network satisfaction^26^, packing around the networks, secondary structure shape complementarity and local distance difference test (LDDT) scores from the deep learning framework DeepAccNet^27^.

**Fig. 2:**
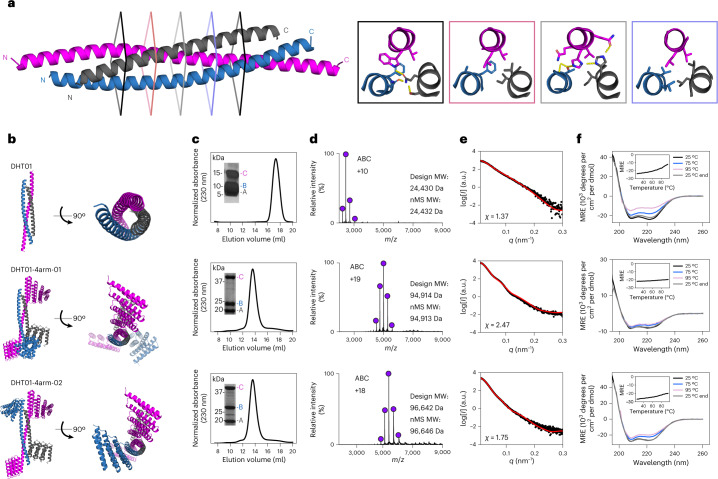
Experimental characterization of designed single-helix heterotrimers. **a**, Colored cross-sections across the coiled coil heterotrimer (DHT01) show core packing across the inner five heptads making up the shared ABC interface, with hydrogen bond networks and hydrophobic packing highlighted. **b**, Design models of DHT01 and two four-arm fusions, colored by chain (dark gray for chain A, blue for chain B and magenta for chain C) and shown in cartoon representation. **c**, Superose 200 chromatograms and SDS-PAGE gels (inset, with the locations of molecular weight markers) for DHT01 (top), DHT01-4arm-01 (middle) and DHT01-4arm-02 (bottom), showing three chains eluting at a monodisperse peak. **d**, nMS results for DHT01 (top), DHT01-4arm-01 (middle) and DHT01-4arm-02 (bottom), showing only the ABC heterotrimer forming. MW, molecular weight. **e**, SAXS profiles for DHT01 (top), DHT01-4arm-01 (middle) and DHT01-4arm-02 (bottom), indicating a good quality of fit (*χ*) between the respective design models (red lines) and experimental data collected (black dots). **f**, Circular dichroism spectra for DHT01 (top), DHT01-4arm-01 (middle) and DHT01-4arm-02 (bottom) at 25 °C (black), 75 °C (blue) and 95 °C (pink) before cooling and after cooling to 25 °C (gray), with thermal melting curves (inset) measured at 222 nm. MRE, mean residue ellipticity. Uncropped gel images for **c** are available as source data. Source data

We obtained genes encoding 20 coiled coil designed heterotrimers (DHTs) in a tricistronic *Escherichia coli* expression vector with one chain having a 6xHis-tag and a second chain having Strep-tag II, expressed the proteins and purified them by immobilized metal affinity chromatography (IMAC) or Strep-Tactin pull-down. All of the designs were soluble and, for six designs, all three components were observed by liquid chromatography–mass spectrometry (LC-MS) after both pull-down approaches (Strep-tag II was used in these first experiments to assess chain dependency in the pull-down experiments, but since all three components were present by sodium dodecyl sulfate polyacrylamide gel electrophoresis (SDS-PAGE) and LC-MS using both the His-tag and Strep-tag, the Strep-tag was omitted from subsequent designs for simplicity). Of these, five eluted as monodisperse peaks by size exclusion chromatography (SEC), but only one design (DHT01) was an exclusive ABC heterotrimer by native mass spectrometry (nMS) (Fig. 2d) and had good agreement with the design model via small-angle X-ray scattering (SAXS) (Fig. 2e and Supplementary Table 1). DHT01 partially unfolds at higher temperatures by circular dichroism, but following arm fusions (see below) is thermostable up to 95 °C (Fig. 2f).

A helical wheel representation of DHT01 (Extended Data Fig. 1) over the shared seven-residue (heptad) interface between all three chains shows that position g is fully nonpolar on chain B and mixed (nonpolar and polar) on chain A, while being fully polar on chain C. This differs from a previously designed heterotrimeric coiled coil solved by X-ray crystallography^3^, which used either glutamate or lysine at positions e and g to dictate specificity. To determine whether nonpolar residues are actually needed at position g, a variant was constructed (Extended Data Fig. 1) with serine, threonine or glutamate mutations on chains A and B. In the IMAC pull-down, chain B was not present stoichiometrically, suggesting that nonpolar residues at g are necessary for DHT01 assembly. Success in generating specificity from purely ionic interactions in the previously determined coiled coil crystal structure could be due to the much smaller size of that construct (each peptide was 4.5 heptads long) and the absence of polar residues at typical solvent-exposed b and c positions (alanine is present instead) to reduce the likelihood of undesired competitive ionic interactions within each chain. This design principle, clearly successful at the peptide scale, could lead to solubility issues in cells and lower robustness to downstream modifications needed for higher-order building, although we have not tested this directly. In our case, DHT01 specificity is probably derived from hydrophobic and hydrogen bond network packing across the a, d and g positions, supplemented by favorable electrostatic interactions across the three chains.

### Extending an ABC coiled coil with repeat protein arms

To determine whether DHT01 could serve as an organizing hub for larger protein assemblies, monomeric designed helical repeat (DHR) proteins^28^ were rigidly fused onto available (N and C) termini using the Rosetta HelixFuse^29,30^ protocol; we refer to each rigid fusion as an arm throughout the text (Fig. 1b). Repeat proteins are an attractive choice for fusion because they can be extended or contracted simply by adding or removing repeat units, and hence allow for considerable design plasticity. Genes encoding two three-arm constructs, four four-arm constructs and one five-arm construct were expressed and purified like DHT01. All designs were soluble, but only three designs had three equimolar components by SDS-PAGE gel in both pull-down experiments. Two four-arm heterotrimers retained exclusive ABC heterotrimer assembly via SEC, nMS, SAXS and circular dichroism; the armed constructs are more thermostable than the original coiled coil heterotrimer at 95 °C (Fig. 2b–f). The ability of the ABC heterotrimer to support rigid repeat protein fusions suggests that the base coiled coil construct folds as designed and provides new connection points for downstream nanomaterials design.

### Design and characterization of ABC helical bundles and arms

To explore the ability to design heterotrimers with larger interfaces available for installing hydrogen bond networks, we extended our computational approach to helical hairpin units. We experimented with two approaches: first, sampling superhelical parameters for all six helices at once (see Methods); and second, making the search more tractable by first sampling parameters for four of the helices, filtering and then adding on the two remaining helices (Fig. 1a). In this second approach, the supercoil radius (*R*), helical phase and *Z* offset (*Z*_off_) were sampled first for only the three inner helices (at superhelical phases 0°, 120° and 240°) and one outer helix (at 60°), then Monte Carlo HBNet^22–24^ was used to search for a four-helix network with at least one tyrosine or tryptophan. For backbones that passed this criteria, *R*, Δɸ_1_ and Z_off_ of an additional fifth helix placed at supercoil phase 180° were sampled and Monte Carlo HBNet was used to search for hydrogen bonds involving this new helix and three of the already placed inner helices. Subsequently, the sixth helix was added at supercoil phase 300° and Monte Carlo HBNet was again used to search for hydrogen bond networks spanning the first three inner helices and the new sixth helix (Fig. 1a). Rosetta combinatorial sequence design calculations were carried out on the resulting helical bundle backbones, keeping the HBNet residues fixed as described above for the coiled coil heterotrimers. Designs with exposed hydrophobic patches were removed using the Rosetta-integrated Developability Index^31^ SAP (spatial aggregation propensity) filter to prevent sticky mis-assemblies and aggregation. To create the ABC heterotrimer, an inner and outer helix were connected with a short loop in either a clockwise or counterclockwise orientation, with loops all on the same side or on opposite ends of the heterotrimer (see Methods).

**Fig. 3:**
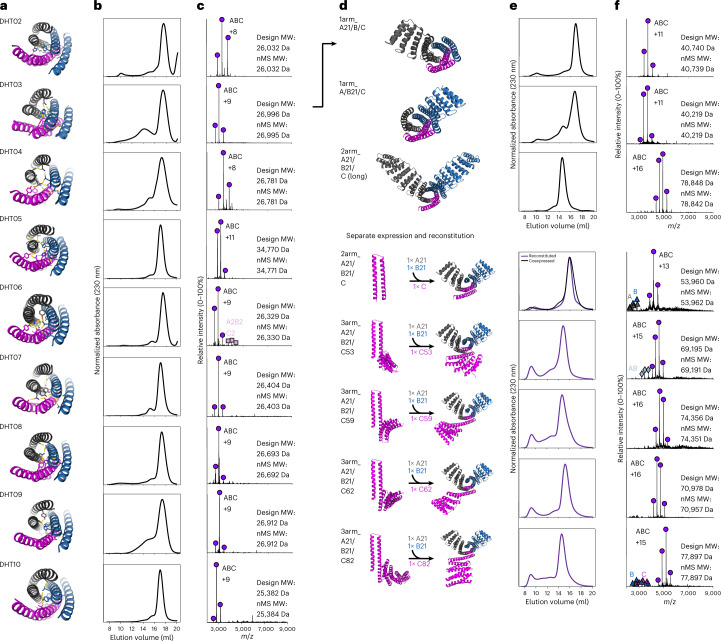
Experimental characterization of designed helical hairpin heterotrimers. **a**, Design models for heterotrimers, colored by chain and shown from a top-down view with a cartoon representation and with hydrogen bond networks in stick representation. **b**,**c**, Monodisperse SEC traces (**b**) and nMS results (**c**) for (from top to bottom) DHT02, DHT03, DHT04, DHT05, DHT06, DHT07, DHT08, DHT09 and DHT10, showing only the ABC heterotrimer forming in almost all cases. **d**, Design models for DHT03 one-, two- and three-arm fusions, colored by chains and shown in a cartoon representation. **e**,**f**, SEC (**e**) and nMS (**f**) for the designs shown in **d**, indicating that the ABC heterotrimer is still present. Components for 2arm_A21/B21/C and subsequent three-arm fusions were separately expressed and individually purified, mixed at a 1:1:1 ratio and then annealed to reconstitute the ABC heterotrimer.

Genes encoding 85 heterotrimers in a tricistronic expression vector were obtained and the proteins were expressed in *E. coli* and purified via IMAC with only one chain having the 6xHis-tag. Nine of the designs (Fig. 3a) had monodisperse SEC peaks (Fig. 3b), were almost exclusively ABC by nMS (Fig. 3c) and had good SAXS fits to the design models (Supplementary Fig. 1 and Supplementary Table 1). Circular dichroism measurements for DHT02–04 showed they were helical and thermostable as expected (Supplementary Fig. 2). Eight of the designs are parallel heterotrimers, while DHT09 is an antiparallel heterotrimer. Twelve additional designs had all three components present by LC-MS but had some heterogeneity (ABC plus other alternative species) in nMS. For three other designs, only the ABC heterotrimer was determined to form by nMS (when the respective SEC fraction was analyzed) but the heterotrimer SEC peak was preceded by soluble aggregate that would make building with these constructs difficult downstream.

**Extended Data Fig. 2:**
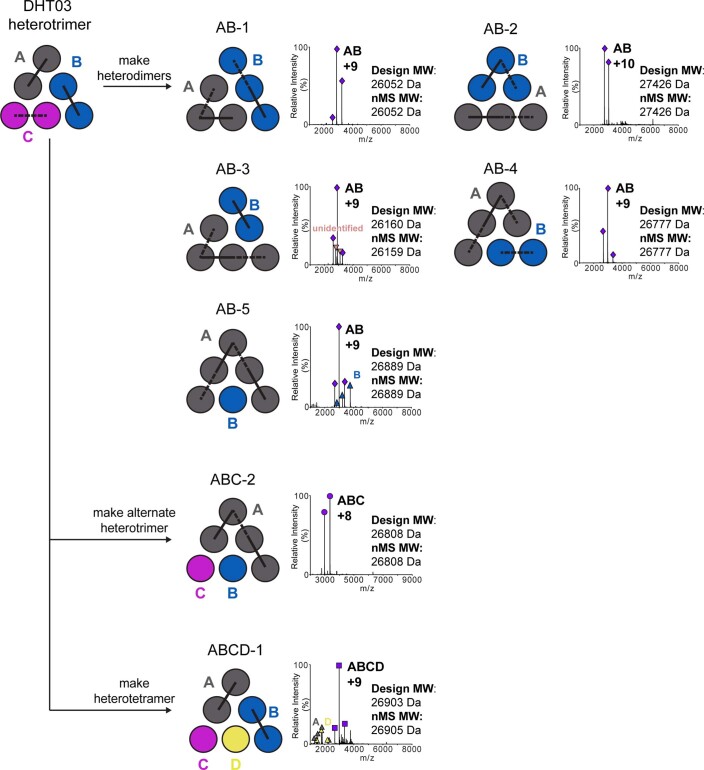
DHT03 can undergo different loop connectivities to form alternate desired oligomers. Different loop closures were applied to DHT03 to create a total of five heterodimers, one alternate ABC heterotrimer, and one ABCD heterotetramer, as determined by native mass spec. The alternate heterotrimer and ABCD heterotetramer were made by removing the existing loop on the chain C helical hairpin, resulting in single helices for each chain. Black solid lines represent loops facing all on the same side, while dashed lines represent loops on opposite ends of termini.

We explored the dependence of assembly of the six helix bundles on the connectivity between the helices by changing the positions of the loops across different helices and adding or deleting loops to create heterotetramers and heterodimers, while keeping the rest of the interface intact. We chose DHT03 as a representative example and found that five heterodimers, one alternative ABC heterotrimer and one heterotetramer assembled specifically, as determined by nMS (Extended Data Fig. 2). The ability to sustain alternative loop closures increases the number and types of heteromeric building blocks available for future nanostructure assembly applications: from just one base design, this generates a family of structures with chain termini available for functionalization through fusion in different locations, and that can bring either two, three or four distinct chains together.

**Extended Data Fig. 3:**
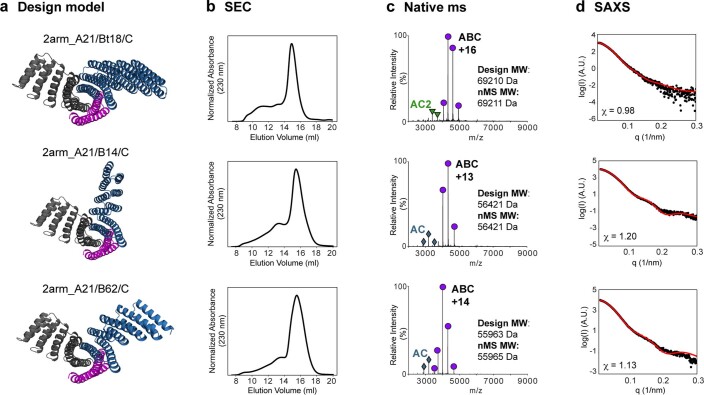
Hierarchical building with DHT03 heterotrimer base. **a** Three more 2-arm constructs built off of 1arm_A21/B/C are shown in a cartoon representation, colored by chain. Curved and straight DHRs were used as input for rigid helical fusion. **b** These constructs had monodisperse peaks by SEC, **c** determined to be ABC by native mass spec, and **d** the proposed design models (red line) had good agreement to experimental (black dots) SAXS data (Supplementary Table 1).

Next, we explored the potential of these larger heterotrimers to serve as nanomaterial-organizing centers using the rigid fusion of repeat protein arms approach described above. We tested these arms sequentially to evaluate the effect of each fusion independently on ABC heterotrimer formation. Using this strategy, we found that, of the first four helical bundle heterotrimers shown in Fig. 3, two were able to sustain three-arm extensions. We illustrate this with DHT03 in Fig. 3d–f and Extended Data Fig. 3 (fusion data for DHT05 are provided in Supplementary Fig. 3). Ten one-arm fusions to DHT03 were tested via tricistronic expression, with two eluting with monodisperse peaks by SEC and forming exclusive ABC heterotrimers by nMS (Fig. 3f, rows 1 and 2). Subsequent two-arm fusions were made by combining the two working one-arm fusions and testing nine more new fusions to chain B while keeping chain A constant. Eight out of the ten designs tested had three equimolar bands by gel; six designs had a distinct heterotrimer SEC peak present with the ABC species detected by nMS; and four had a very good fit to the design model via SAXS (Fig. 3f (row 3), Extended Data Fig. 3 and Supplementary Fig. 1).

### Arms can be expressed separately and reconstituted to ABC

To facilitate downstream higher-order assembly design, we investigated whether the ABC heterotrimer could be reconstituted from separately expressed individual chains. Because of the hydrophobic nature of the core, each chain of the heterotrimer can self-associate when expressed separately. We reasoned that the designed ABC heterotrimer state would probably be lower in free energy than possible off-target homo-oligomeric species, and hence that heat annealing could promote assembly to the design target state. To test this idea, equimolar amounts of individually purified A, B and C of DHT03_2arm_A21/B21/C were mixed together and annealed (see Methods) to allow these interfaces to reassemble in the presence of all components. A monodisperse SEC peak containing all three chains by nMS (Fig. 3f) was observed at the same elution volume as the co-expressed tricistronic version (Fig. 3e), indicating successful reconstitution of the heterotrimer from independently expressed proteins. To explore the use of this approach for building, four different repeat protein fusions to chain C were annealed in the same manner and all four three-arm heterotrimers were successfully reconstituted. The successful reconstitution of these ABC assemblies, following the independent expression and purification of each chain and explicit mixing at a 1:1:1 ratio, enables their use in conjunction with other de novo proteins to mediate the specific assembly of multi-component assemblies (it also overcomes the disadvantage of co-expression in which the levels of each chain cannot be precisely controlled, resulting in stoichiometric imbalances that could complicate proper assembly). Independent expression also has considerable advantages over tricistronic expression from a gene synthesis perspective as DNA becomes much more difficult and expensive to synthesize with increasing construct size.

**Extended Data Fig. 4:**
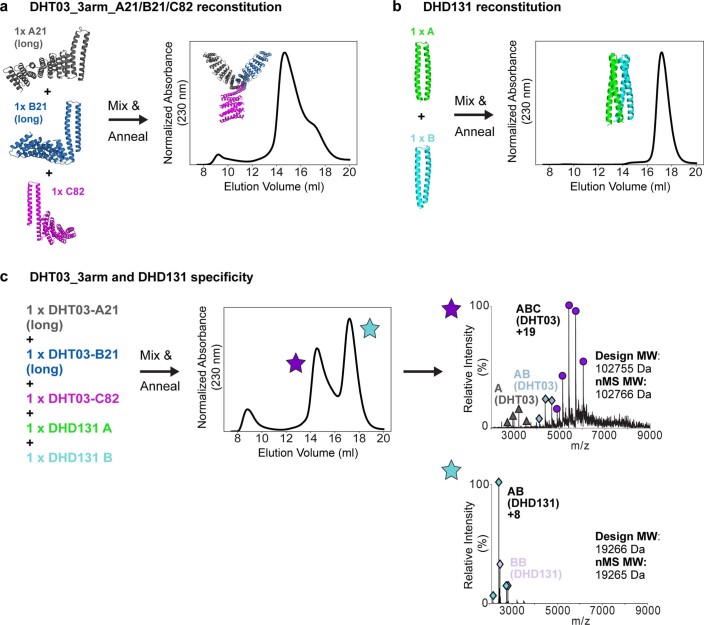
Separate expression and reconstitution via annealing for a previously designed heterodimer (DHD) and its specificity when mixed with DHT03. **a** Three chains for DHT03_3arm_A21/B21/C82 were separately expressed and individually purified, mixed together at an equimolar ratio (10uM each), annealed, and run through SEC. A peak corresponding to the heterotrimer is observed. **b** Two chains for DHD131 were separately expressed and individually purified (each with noticeably low solubility), mixed together at an equimolar ratio (10uM each), annealed, and run through SEC. A monodisperse peak corresponding to the heterodimer is observed. **c** All five chains for DHT03_3arm and DHD131 were mixed together at an equimolar ratio (10uM each), annealed, and run through SEC with two peaks observed matching the original controls in **a** and **b**, respectively. The main fraction from each peak was analyzed by native mass spec, which determined the correct reconstitution of the DHT03 heterotrimer and DHD131 heterodimer, without the presence of a chain from the other hetero-oligomer being tested.

To test whether the DHT03 components could properly assemble in the presence of helical hairpin-containing chains involved in other oligomers, as would be the case for more complex assemblies, we chose DHD131, a designed helical hairpin unit heterodimer containing buried hydrogen bond networks^24^. We found that our separate expression and reconstitution approach via annealing succeeded with this design: chains A and B of DHD131 could be separately expressed and purified, and when the proteins were mixed together at an equimolar ratio and annealed, a monodisperse heterodimer peak was observed by SEC (Extended Data Fig. 4). We separately expressed and individually purified the two chains of the DHD131 heterodimer and the three chains of the DHT03 heterotrimer, mixed them at a 1:1:1:1:1 ratio and carried out annealing and SEC as described above. The two major species were found by nMS to be the DHD131 heterodimer and the DHT03 heterotrimer (Extended Data Fig. 4). Some DHD131 BB homodimers were also detected, as was observed in previous nMS analyses of the heterodimer alone^24^. Thus, the heterotrimer chains can come together to form the intended ABC species even in the presence of potentially confounding additional helical hairpins. The ability to simultaneously assemble multiple hetero-oligomers from individual chains without interference opens the door to the construction of diverse nanostructures with distinct multichain hubs.

### X-ray crystal structures of DHT03

**Fig. 4:**
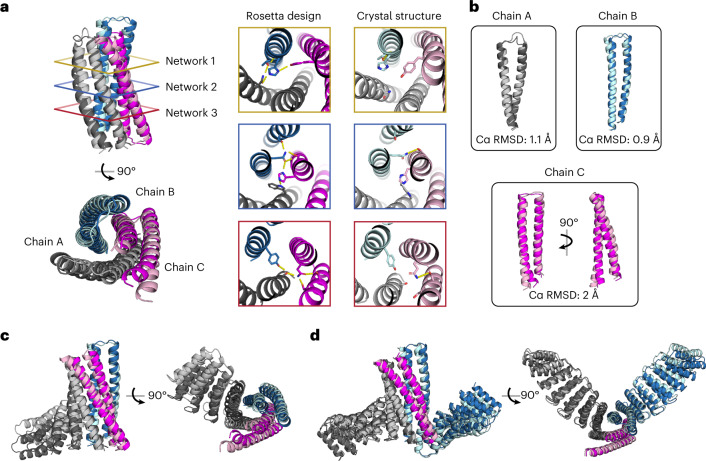
Crystal structures of DHTs. **a**, The design model (darker colors) for the DHT03 base heterotrimer is aligned to the crystal structure (lighter colors), shown in cartoon representation from a side and top-down view. Colored cross-sections to the right show hydrogen bond networks in the design versus the crystal structure. **b**, Independent chains show Cα RMSD alignment between the design and crystal structure, with the largest deviation in chain C. **c**, The design model (darker colors) of the DHT03 one-arm heterotrimer is aligned to the crystal structure (lighter colors), shown in cartoon representation from a side and top-down view. **d**, The design model (darker colors) for the DHT03 two-arm heterotrimer with propagated repeats, after being informed by **c**, is aligned to the crystal structure (lighter colors).

**Extended Data Fig. 5:**
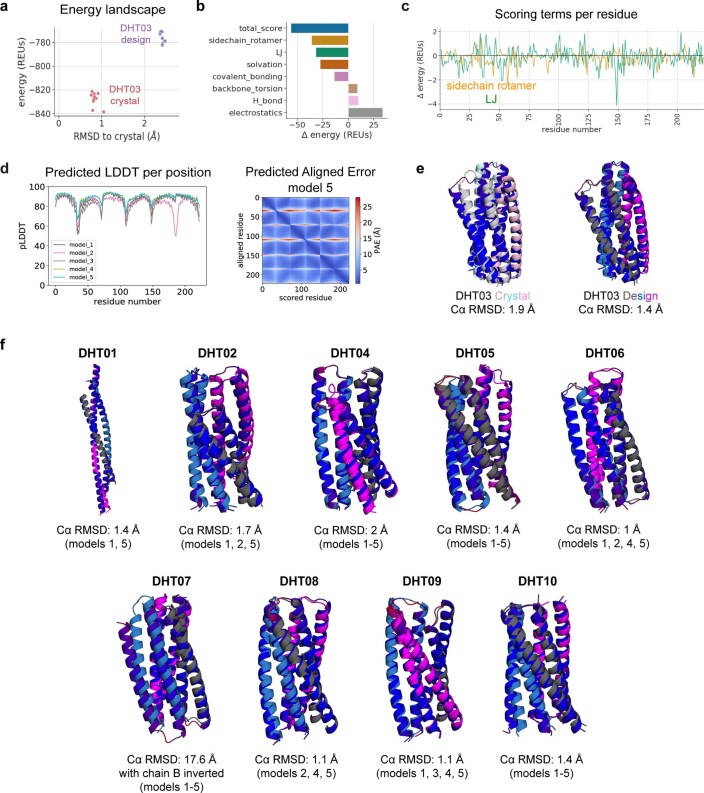
Analyzing DHT03 heterotrimer with Rosetta and using structure prediction to determine base heterotrimer assembly. **a** Computed energy landscape for DHT03, showing 10 replicate relaxes for the crystal structure (purple dots) and the original Rosetta design model (red dots). The RMSD to the un-relaxed crystal structure is computed across all Cα and plotted against Rosetta energy in Rosetta Energy Units (REUs). The relaxed design models have higher energies than the relaxed crystal structures. **b** The best-scoring relaxed crystal structure is compared to the best-scoring relaxed design model, showing energy differences for different score terms in Rosetta’s energy function. Negative values indicate that the score term favors the relaxed crystal structure. **c** Sidechain_rotamer and LJ terms are plotted on a per-residue level, with negative energy differences indicating that preference for the relaxed crystal structure arises from a summation of many small effects. **d** DHT03 predicted LDDT per residue is plotted for all 5 AlphaFold-Multimer^32^ models; Predicted Aligned Error (PAE) heat map for model 5 indicates low predicted Å error between pairs of residues across the interface. **e** Model 5 prediction (colored by pLDDT using a red-blue color spectrum) is aligned to DHT03 crystal structure (left) and the Rosetta design model (right), with a slightly better agreement to the design. **f** Single sequences for heterotrimer bases presented were used as input for AlphaFold-Multimer with the number of recycles set to 3. A representative prediction (colored by pLDDT using a red-blue color spectrum) with the lowest Cα RMSD is aligned to the design models colored by chain. Models predicting structures close to the designs are listed in parenthesis, with scores for AlphaFold confidence metrics pLDDT and pTM for all 5 AlphaFold-Multimer models reported in Supplementary Table 2 (the highest pLDDT and pTM scores indicate good agreement to DHT04, 05, 06, 08, 09, 10 design models). All 5 AlphaFold-Multimer models for DHT07 predict an inverted chain B.

We succeeded in determining three high-resolution structures for DHT03: the original base construct, a one-arm version (1arm_A21/B/C) and an elongated two-arm version (2arm_A21/B21/C long). The first crystal structure (Fig. 4a) was determined to 2.35 Å resolution (Table 1). The design model (shown in darker colors) has an overall 2 Å Cα root mean square deviation (RMSD) agreement to the crystal structure (shown in lighter colors) with the largest deviations upon individual chain superposition in chain C (Fig. 4b). Figure 4a shows colored cross-sections of each hydrogen bond network in the design model compared with the crystal structure. Many of the hydrogen bonds in the design model are not present in the crystal structure, but the overall placement of these residues is relatively close in space to the design model and the ABC heterotrimer still assembles with buried polar groups in the core (water molecules were not detected in Fo–Fc maps, but we cannot entirely rule them out as the overall completeness of the dataset was ~75%). The placement of these residues also appears to be effective in specifying orientation as the chain C orientation in the design model matches that of the crystal structure. There are two possible explanations for the deviations in the hydrogen bonding between the crystal structure and design model. The first is that optimization of nonpolar packing in the actual structure distorts the protein slightly so that many of the designed hydrogen bonds do not form. In support of this, the crystal structure has lower Rosetta-computed energy than the design model (Extended Data Fig. 5a–c) due to improvements in sidechain rotamer preferences, Lennard–Jones/van der Waals interactions and solvation energies. The second possibility is that, in solution or neighboring low-energy states, small backbone adjustments allow for more or all of the designed hydrogen bonds to form (Rosetta may not accurately capture the energetic cost of buried unsatisfied hydrogen bonds). In any event, a lesson from this structure is that more extensive sampling around the designed conformation in the design process would be useful to determine whether nonpolar packing and hydrogen bond networks favor the same state; because of the short-range nature of the hydrogen bond and the strong orientational constraints, even small distortions away from the design model can disrupt hydrogen bond networks.

**Table 1:** Data collection and refinement statistics

	DHT03_2arm_A21/B21/C long	DHT03	DHT03_1arm_A21/B/C
Data collection			
Space group	*P*1	*P*1	*P*1
Cell dimensions			
*a*, *b*, *c* (Å)	59.47, 68.05, 98.99	37.25, 39.92, 50.42	55.73, 73.5, 97.28
*α*, *β*, *γ* (°)	106.461, 108.516, 93.086	88.974, 105.789, 115.319	73.422, 82.919, 80.781
Resolution (Å)	45.87–3.35 (3.47–3.35)^a^	48.21–2.10 (2.175–2.1)^a^	49.01–3.35 (3.47–3.35)^a^
Total observations	35,399 (3,655)^a^	22,444 (919)^a^	37,099 (3,840)^a^
Unique reflections	18,621 (1,887)^a^	11,814 (500)^a^	19,545 (1,994)^a^
Redundancy	1.9 (1.9)^a^	1.9 (1.8)^a^	1.9 (1.9)^a^
Completeness (%)	76.72 (60.34)^a^	73.95 (24.42)^a^	84.33 (71.67)^a^
*R*_meas_	0.09258 (1.532)^a^	0.0573 (1.003)^a^	0.1078 (1.082)^a^
*I/σ*(*I*)	6.5 (0.78)^a^	8.68 (0.95)^a^	6.88 (1.01)^a^
*CC*_1/2_	0.998 (0.554)^a^	0.994 (0.646)^a^	0.998 (0.431)^a^
Refinement			
Resolution (Å)	45.87–3.35	48.21–2.10	49.01–3.35
Reflections (work)	18,614	11,814	19,543
Reflections (test)	751	1,094	1,772
*R*_work_/*R*_free_ (%)	29.47/32.69	22.61/27.26	24.91/30.51
Average B-factor (Å^2^)	133.68	75.62	98.69
Number of atoms			
Protein	5,365	1,913	11,098
Water	0	5	0
Ramachandran^b^			
Favored (%)	100	98.65	98.91
Outliers (%)	0	0	0.15
Rotamers^b^			
Favored (%)	96.67	97.31	96.47
Outliers (%)	0.53	0	0.25
RMSDs			
Bond lengths (Å)	0.002	0.002	0.002
Bond angles (°)	0.88	0.38	0.37
Molprobity^b^			
Molprobity score	1.32	1.19	1.31
Percentile	100th	100th	100th
Clashscore	5.91	4	5.68
Percentile	100th	99th	100th
PDB ID	7UPP	7UPO	7UPQ

^a^Numbers in parentheses refer to the highest-resolution shell.

^b^As reported by MolProbity^37^.

The crystal structures of the one-arm (Fig. 4c; biophysical data in Fig. 3d–f, row 1) and elongated two-arm (Fig. 4d; biophysical data in Fig. 3d–f, row 3) fusions were both determined to 3.35 Å resolution (Table 1). Over the core heterotrimer in both structures, the backbones are very similar to that of the base heterotrimer crystal structure, but the resolution is not high enough to determine the state of the designed hydrogen bond networks. The designed junctions between the repeat protein arms and the core bundle are recapitulated in the crystal structures and hold the repeat arms rigidly in close to the designed orientations. Deviations between the crystal structure and design model in the repeat protein arms increase with increasing distance from the central bundle due to lever arm effects arising from the compounding of small orientational differences in the structures of the individual repeats^28^. These structures show that the DHTs can serve as rigid interaction hubs in asymmetric assemblies, which have been more difficult to design than symmetric ones.

We were not able to crystallize the remaining nine base heterotrimers and turned to protein structure prediction to supplement the SEC, nMS and SAXS data presented above. For DHT03, four out of five AlphaFold-Multimer^32,33^ models generated heterotrimeric structures similar in overall topology; the predicted structures are closer to the Rosetta design model than the crystal structure when aligned across all Cα atoms (Extended Data Fig. 5d,e). For eight of the other nine heterotrimer designs, at least two out of five AlphaFold-Multimer^32,33^ models were within 2 Å Cα RMSD of the design models (Extended Data Fig. 5f, Supplementary Table 2; the physically based Rosetta and deep learning-based AlphaFold should be largely orthogonal so this level of agreement constitutes somewhat independent validation). While not as definitive as crystal structures, the combination of structure prediction and biophysical data support the design models.

### Core residues with polar groups are needed for specificity

**Extended Data Fig. 6:**
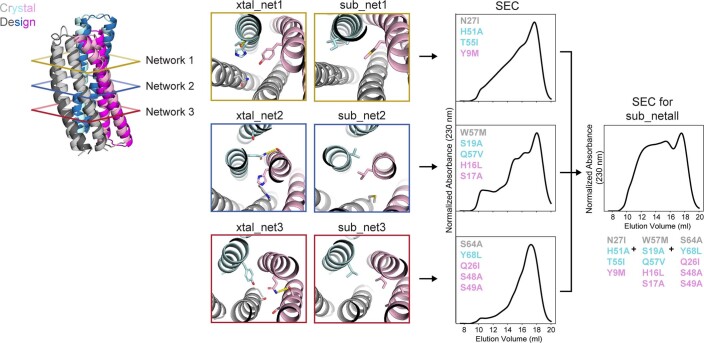
Systematic replacement of intended network residues in crystal structure indicates their importance for mediating ABC specificity. Colored cross-sections of the helical bundle base heterotrimer show hydrogen bond networks in the crystal structure (left), compared to (middle) Rosetta-preferred nonpolar residue substitutions, which shows broad SEC output (right) indicating ABC heterogeneity. The amino acid substitutions are shown in each SEC spectra and colored by the respective crystal structure coloring scheme: light gray for chain A, light cyan for chain B, and pink for chain C. The combination of all 3 replacements which results in a fully hydrophobic core, ‘sub_netall,’ shows the messiest SEC chromatogram seen for this design.

To better understand the importance of the buried polar groups for exclusive ABC heterotrimer assembly in DHT03, the residues intended to form hydrogen bond networks were systematically replaced. Starting from the crystal structure, residues involved in each network were repacked with nonpolar residues using Rosetta, while keeping the remaining residues intact. We refer to these as sub_net1, sub_net2 and sub_net3, while the combination of all of these substitutions was called sub_netall (Extended Data Fig. 6). These four constructs were purified via the same IMAC pull-down approach as the parent heterotrimer design. In all four cases, the A, B and C components were present in the eluate by LC-MS but the SEC spectra had broad diffuse peaks, suggesting heterogeneity in the assemblies. The broadest SEC trace was observed for sub_netall with the entirely hydrophobic core. These results suggest that the buried polar residues contribute to structural specificity.

### Using ABC arms to build multi-component cyclic assemblies

**Fig. 5:**
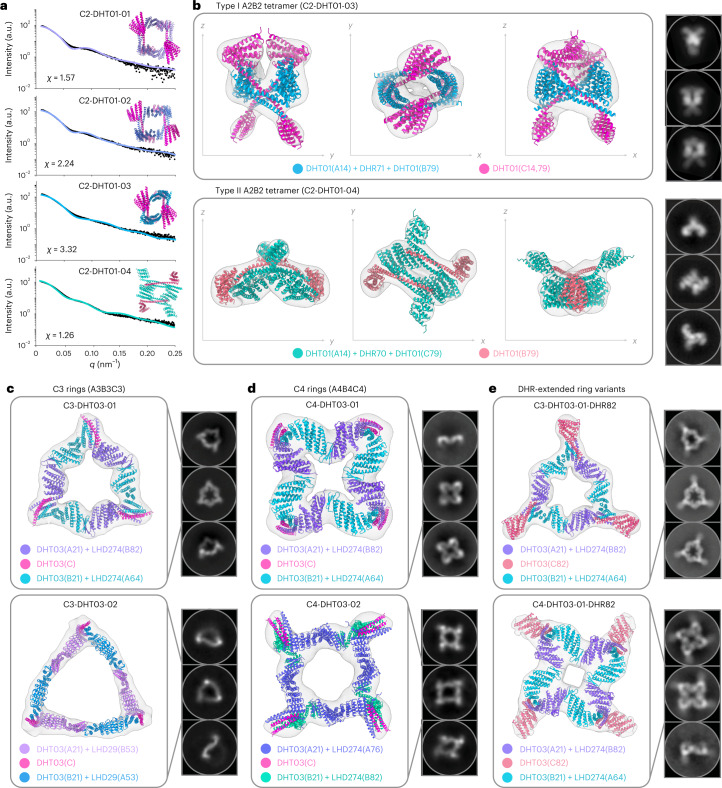
Recursive design of higher-order assemblies. **a**, SAXS profiles indicate a good quality of fit (*χ*) between the design model (line) and experimental scattering data (black dots) for four A2B2 tetramers: three type I tetramers (C2-DHT01-01, C2-DHT01-02 and C2-DHT01-03) and one type II tetramer (C2-DHT01-04). In panels **b**-**e**, design models are shown with different chains in different colors, superimposed on nsEM reconstructed 3D maps, with 2D class averages on the right. **b**, a type I A2B2 heterotetramer (C2-DHT01-03; top) and a type II A2B2 heterotetramer (C2-DHT01-04; bottom). **c**, Characterization of two A3B3C3 cyclic designs: C3-DHT03-01 (top) and C3-DHT03-02 (bottom). **d**, Characterization of two A4B4C4 cyclic designs: C4-DHT03-01 (top) and C4-DHT03-02 (bottom). **e**, Fusion accessibility of the a third chain enables further design opportunities. Versions of C3-DHT03-01 and C4-DHT03-01 with DHR82 fused to chain C of DHT03.

**Extended Data Fig. 7:**
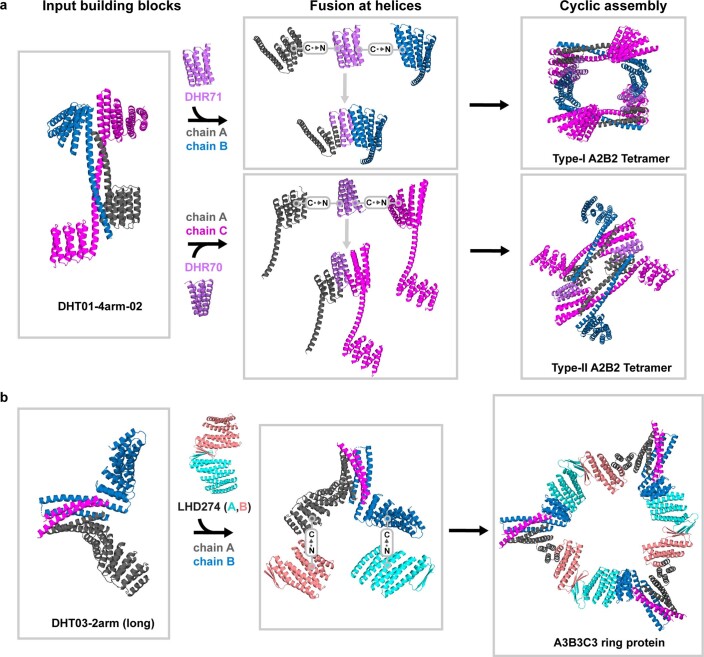
Building block connections during cyclic fusion of A2B2 tetramers. **a** The cyclic design strategy for A2B2 tetramers involved splicing two types of chain combinations from DHT01-4arm-02. For type-I A2B2 tetramers (top), the C-terminal DHR-arm of chain A is fused to the N-terminus of a different DHR repeat segment, which then has its C-terminus fused to the N-terminal DHR-arm of chain B. For type-II A2B2 tetramers (bottom), the C-terminal DHR-arm of chain A is fused to the N-terminus of a different DHR repeat segment, which then has its C-terminus fused to the N-terminal DHR arm of chain C. Colors indicate the regions in the cyclic assemblies that come from each constituent chain from the input building blocks. **b** The cyclic design strategy for ring-proteins made from the DHT03 helical bundle heterotrimer. Both DHR-arms in DHT03_2arm_A21/B21/C have only N-termini accessible for fusion, necessitating two separate fusion steps to form the cyclic structures, with each fusion step joining one of DHT03’s DHR-extended arms to a C-terminal DHR chain of an LHD heterodimer, which possessed fusion-accessible C-termini on both of its chains.

To investigate the potential of the ABC heterotrimers to serve as multichain connection hubs in larger designed nanostructures, we employed the WORMS^29,34^ software, which searches over very large numbers of possible rigid fusions between building blocks to build up user-specified architectures. In a first round of architecture design, the chains of the four-arm coiled coil ABC heterotrimers from Fig. 2 were fused with a library of DHR proteins to generate closed rings with different cyclic symmetries. As illustrated in Fig. 1c, there are two ways to connect two of the three DHT01-4arm-02 chains to form closed cycles: (1) fusion between the single DHR arms on chains A and B; or (2) fusion between the chain A DHR arm and one of the two chain C DHR arms. These generate type I and type II closed assemblies, respectively. The placement of the original heterotrimer chains in these assemblies is indicated schematically in Fig. 1c and Extended Data Fig. 7a. Twelve designs were experimentally tested via bicistronic expression. Of these, four A2B2 heterotetramers had good agreement via SAXS (Fig. 5a and Supplementary Table 1) and rings were evident in negative stain electron microscopy (nsEM), with three-dimensional (3D) reconstructions obtained for two of the constructs (Fig. 5b).

To expand the range of geometries, and to enable nanostructure assembly from individual components, we sought to design a second round of cyclic structures using the propagated two-arm ABC heterotrimer crystal structure together with alpha/beta (LHD) heterodimers^35^, as shown schematically in Fig. 1c and Extended Data Fig. 7b. This combination of two different types of de novo hetero-oligomers in one closed structure is challenging because even slight changes in the angles of newly designed fusion points can have a substantial impact on the resulting geometries. Genes were obtained for five A3B3C3, two A4B4C4 and three A5B5C5 rings. Ring structures were observed for six designs by nsEM; two designs displayed a mixture of C3- and C4-symmetric states, but the remaining four samples (two A3B3C3 and two A4B4C4 rings) were homogeneous (Supplementary Fig. 4). 3D reconstructions for three of these four rings revealed cyclic symmetry one degree lower than the computational designs (C3-DHT03-02 formed an A3B3C3 ring as intended). Assembly of smaller rings results in less loss of translational entropy; this probably results from small inaccuracies in the design models of the components lacking crystal structures and internal flexibility of the repeat protein connectors. Regenerating the design models using the experimentally observed stoichiometries (Supplementary Fig. 5) yielded structural models that closely fit the electronic density maps (Fig. 5c,d).

As is evident from the images in Fig. 5c,d, the cyclic structures retain one of the original heterotrimer helical hairpins in each corner; these short, outward-facing protein chains provide fusion points for further elaboration of higher-order symmetric assemblies. To evaluate the potential for building up larger nanostructures using the rings as hubs, and to explore the modularity of the designed building blocks, we replaced the original outward-facing chain C helical hairpin with the chain C DHR82 fusion (as it was the largest four-repeat DHR) from Fig. 3d, row 8. This was done for two designs (C3-DHT03-01 and C4-DHT03-01), with chain C replaced by fusion C82 through expressing and purifying the three components separately, mixing them in equal molar quantities and reconstituting using heat annealing. The primary-peak fractions from SEC were collected and inspected by nsEM, and rings with DHR82-extended arms were observed for both C3-DHT03-01 and C4-DHT03-01. The micrographs and reconstructed 3D electronic density maps (Fig. 5e) displayed clear arms extending from the rings, indicating successful assembly of symmetric complexes with branching capabilities at this terminus.

## Discussion

We show that computational design can create cooperative ABC heterotrimers that can assemble as free-standing helical units or as hubs in larger designed assemblies. Their small base size and high soluble expression make them useful for biological scaffolding applications involving the recruitment or display of three different proteins. The ability to sustain rigid helical fusions to monomeric repeat proteins enables the incorporation of arms that can be extended to provide three new elongated connection points. We show that the heterotrimers can be used as interaction hub building blocks between chains in larger closed structures—A2B2 heterotetramers, A3B3C3 nonamers and A4B4C4 dodecamers—generated through geometry-aware rigid fusion to themselves and other designed proteins. These new assemblies can be built out recursively, unlike previously designed rings^35^, as they have outward-facing chains with two or more free termini. The number and orientational accessibility of the chain termini found in both the base heterotrimers and the higher-order assemblies may enable the display of multiple distinct functional domains for signaling and other potential applications. As illustrated by the larger ring designs, the modularity and orthogonality of the designed protein interfaces make it possible to combine the heterotrimers with other heteromeric building blocks to construct more diverse nanostructures.

Our results also highlight areas for future investigation. The relatively low success rate in achieving pure ABC heterotrimers probably reflects the greater complexity of designing three-sided interfaces compared with two-sided interface design^35^. Our crystal structures, together with the mutational data, pose a fundamental biophysics puzzle. On the one hand, the crystal structure of DHT03 shows that many of the designed hydrogen bond network residues are not making hydrogen bonds due to small local distortions in the structure. Instead, they appear to be buried without making any hydrogen bonds, which is expected to be extremely destabilizing. On the other hand, while DHT03 assembles exclusively to the heterotrimer state, mutants in which the hydrogen bond networks have been substituted by nonpolar residues appear to adopt a range of alternative states, suggesting that the hydrogen bond networks are playing an important role in conferring structural specificity, as in our design conception. It is possible that these residues confer specificity even without making hydrogen bonds, as alternative states could have still higher energies when they are present. Alternatively, there may be very similar states populated in solution in which the designed hydrogen bond networks favoring the designed assembly are formed.

**Extended Data Fig. 8:**
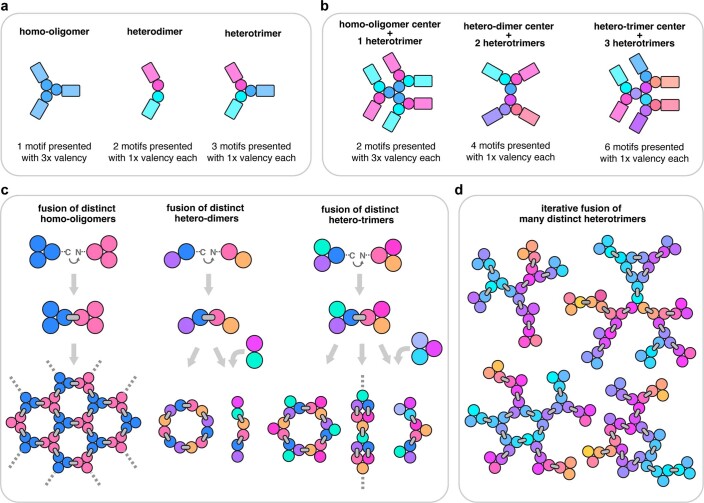
Opportunities and limitations of designs using fusion of multiple distinct oligomers. **a** Functional motifs can be affixed upon oligomeric proteins. Cyclic homo-oligomers enable presentation of motifs with high valency, whereas heterodimers enable presentation of two distinct functional motifs in conjunction. Hetero*trimers* extend such possibilities by enabling the presentation of three distinct motifs at a time. **b** Heterotrimers can provide an increase in valency while also presenting multiple distinct motifs in conjunction by splicing motifs two of their chains, while joining the third chain to a symmetric homo-oligomer. Additionally, the number of distinct motifs that can be presented by these constructs can be increased further by fusing multiple heterotrimers to a central heterodimer, or another central heterotrimer. **c** The geometries resulting from fusion of two symmetric homo-oligomers are limited to regular polyhedral groups (nanocages) or wallpaper groups (2D lattices), as defined by the point-symmetries of their constituent interfaces and the angle between symmetry axes. When using heterodimers to create higher order assemblies through rigid fusion, two fusion-accessible termini are used up with each splice step, limiting the range of designs to chains or rings. In contrast, higher ordered assemblies made through fusion of two or more heterotrimers are not limited to the geometric constraints of homo-oligomers or branching constraints of heterodimers; each rigid fusion step leaves a net increase in the number of fusion-accessible chains, enabling extensive opportunities for subsequent splicing. **d** The iterative fusion of many distinct heterotrimers enables exploration of many new asymmetric constructs, and extensively branching designs that can incorporate many distinct domains. While heterotrimers can be combined with homo-oligomers to create branching symmetric designs, the design of extensively branching asymmetric designs is an avenue that can only be explored using heterotrimeric building blocks.

In previous work, cyclic homo-oligomers and heterodimers have been used to design higher-order materials; the symmetry of the oligomer dictates the type of higher-order geometry that can be constructed^29^. As illustrated in Extended Data Fig. 8, only closed structures with point group symmetries, primarily regular polyhedral nanocages or open regularly repeating lattices can be generated from symmetric homo-oligomeric building blocks, whereas designs containing only hetero-dimeric building blocks are limited to simple bounded assemblies, such as rings or linked chains^29,35^. With modular heterotrimeric building blocks such as those developed in this paper, a much wider range of asymmetric assemblies become accessible, as each additional heterotrimeric interface introduces new centers for asymmetric branching (Extended Data Fig. 8). Design applications for assemblies built from components with cyclic symmetry can be limited by the small number of unique accessible termini for functionalization, whereas—as illustrated by the cyclic rings designed here—the use of heterotrimeric building blocks will generally result in multiple fusion-accessible termini, providing modular design opportunities to build on a common base scaffold (Supplementary Fig. 6). More generally, these heterotrimeric interaction hubs are attractive starting points for nanomaterial applications that require symmetry breaking^36^ to generate more sophisticated protein assemblies.

## Methods

### Computational methods

#### Backbone sampling

For single-helix heterotrimers, three helices were fixed at supercoil phases 0°, 120° and 240° to generate chains A, B and C, respectively. Helix termini were kept in the same direction for a parallel orientation, while the third helix at supercoil phase 240° was inverted for an antiparallel orientation. To create a left-handed coiled coil, the supercoil and helical twist were kept at ideal values of −2.85 and 102.85, respectively. The helical phase (Δɸ_1_) was sampled from −100° to 100° with an interval of 20°. The three helices were sampled at a 6.5–7.5 Å distance (*R*) from the *z* axis with an interval of 0.25 Å. *Z*_off_ was also sampled and kept at 0 for the first helix, but then sampled at −1.5, 0 and 1.5 for each of the second and third helices to account for the rise per residue. All helices were sampled independently across all parameters. Each helix was 77 residues in length.

For the six-helix heterotrimers in the first sampling approach, the supercoil radius (*R*) and helical phase (Δɸ_1_) were sampled independently for parallel backbones, with supercoil phases fixed at 0°, 120° and 240° for the first three inner helices and 60°, 180° and 300° for the remaining three outer helices. If the same parameters from the coiled coil search were applied here for all six helices, more than hundreds of billions of backbones would need to be sampled simultaneously. Instead, the helical phase (Δɸ_1_) was sampled from 0° to 100° with an interval of 20° and the supercoil radius (*R*) was sampled at a 6.5–7.5 Å distance with a 0.5 Å interval for the inner three helices and at 12.5–13.5 Å with a 0.5 Å interval for the outer three helices.

In the second approach, only the first three inner helices and one outer helix were sampled in the first round. The helical phase (Δɸ_1_) was sampled from −100° to 100° with an interval of 20°. The three inner helices were sampled at a 6.5–7.25 Å distance (*R*) from the *z* axis with an interval of 0.375 Å. The fourth outer helix was sampled at a 12.25–13.25 Å distance with an interval of 0.5 Å. *Z*_off_ was kept at 0 for the first helix and sampled at −1.5, 0 and 1.5 for the second, third and fourth helices. The fifth and sixth helices were then each individually sampled across the same three parameters as the fourth helix. Helix termini were kept in the same direction for a parallel orientation, while the third helix at supercoil phase 240° and the sixth helix at supercoil phase 300° were inverted for an antiparallel orientation. Each helix was 35 residues in length.

#### Design of hydrogen bond networks

All polar residues and acidic charged (Asp/Glu) residues were considered during the search. A total of 100,000 Monte Carlo trials were attempted with extra rotamers parsed through to help increase sampling. A minimum of two Trp/Tyr residues were required to be part of the networks. For single-helix backbones, hydrogen bond networks were searched across every other heptad such that a final core N–P–N–P–N–P–N–P–N heptad search pattern would result (N = nonpolar; P = polar). Networks were required to span all three helices and to consist of at least three residues, with a total of three networks across the heterotrimer.

For six-helix backbones resulting from the first parametric sampling approach, hydrogen bond networks were searched across the three middle heptads. Networks were required to span five or six helices, consisting of at least six residues, with at least two networks in total. We required that each network contain at least one tyrosine, tryptophan, aspartate or glutamate. Overall, the Monte Carlo HBNet search was slower due to the increased search space across the middle heptads at all helices, few fully satisfied long networks were found meeting our requirements and, ultimately, Rosetta design packing around six- to ten-residue networks (compared with three- to four-residue coiled coil networks) was more difficult, which led us to focus on the second sampling approach.

For six-helix backbones resulting from the second sampling approach, three hydrogen bond networks were searched for, such that all networks span across the three inner helices and the newly built outer helix. This would yield a core N–P–P–P–N heptad search pattern, in which every helix contributes at least one residue to a hydrogen bond network. We hypothesized that fully hydrophobic heptads above and below the networks would help to keep the hydrogen-bonding residues in place.

#### Rosetta design

Chains B and A for the coiled coil backbone were trimmed by two and four heptads, respectively, resulting in chain A being 49 residues, chain B being 63 residues and chain C being 77 residues long. Two helices of the six-helix backbone (which would ultimately constitute one helical hairpin) were optionally trimmed by one heptad. Both sets of heterotrimer bases underwent packing using RosettaDesign^27^, with six-helix backbones having an additional SAP mover and filter. Constraints on hydrogen bond network residues were placed. The backbones were divided up by layers (core, boundary and surface) with two total packing rounds. A scoring term was also used to enforce at least two phenylalanines at the core. A round of Fast Design calling a Monte Carlo mover was applied to enhance the secondary structure shape complementarity, along with an upweighted short-range hydrogen bond scoring term to maintain proper helical formation. A final minimization and repack of the sidechain rotamers was allowed after removing constraints on network residues.

#### Loops to make helical bundle heterotrimers

Six-helix heterotrimers are closed into three helical hairpin chains in either a clockwise (A–D; B–E; C–F) or counterclockwise (A–F; B–E; C–D) orientation. Short two- to five-residue loops were generated in Rosetta with favorable ABEGO types. Loops were built from either available terminus on each chain, with an option to delete up to three residues or to add two more residues to the existing termini to build off as a starting point. Loops were minimized and filtered by low-fragment RMSD and PSIPRED.

#### Rigid arm fusions to DHRs

Rosetta HelixFuse^29^ was used to rigidly join a library of DHRs to heterotrimer bases by joining the termini of both constructs based on secondary structure overlap; up to a heptad on the heterotrimer was allowed to be deleted, while up to a full single repeat was permitted to be deleted for the DHR. The lowest-scoring RMSD overlap was accepted. A filter was subsequently applied to check for clashes between the two joined proteins to determine residues that needed to be redesigned; RosettaDesign^25^ was used to find optimal residues for the new helix. The best-scoring fusions according to LDDT^27^ and after manual inspection were ordered. For DHT01 fusions, solutions were found at only five of the six available termini.

#### Design of cyclic rings

The generation of cyclic assemblies using WORMS fusion was performed following protocols presented in previous literature^29,34^.

Input building blocks for components (heterotrimers, heterodimers and monomeric DHRs) were curated in a WORMS database file, wherein each entry included specification of the scaffold class, the PDB file path and the range of helical residues up or downstream from the amino (N) and carboxy (C) termini that were accessible as splice sites. Examples of the database file entries for heterotrimers, heterodimers and DHRs are included in Supplementary Note 1.

Within the WORMS software, cyclic symmetry protocols were performed (C2, C3, C4 and C5) such that closure between cyclically propagated copies of the input building blocks could be found by coordinate alignment between residues within the specified fusion-accessible helices from each splice partner. The cyclic protocol flags used to perform these searches in WORMS are specified in Supplementary Note 2.

For rings made from DHT01, the rigidly fused DHR arms were joined at N- and C-terminal helices and an additional DHR repeat motif was used to bridge the two heterotrimer chains. For rings made from DHT03, the rigidly fused DHR arms both possessed N-terminal helices, necessitating their fusion to alpha/beta heterodimers that possessed two C-terminal DHR arms, to close the cyclic geometry.

The outputs from the WORMS algorithm were filtered by three criteria: sequence length, internal clashing and ring closure error; these values for each WORMS output were presented in score files under the fields chain_len, score0 and close_err, respectively. The selected designs were then passed through rigid backbone sequence design using RosettaDesign^26^ to optimize the local sequence around the newly formed helical junctions. The modified positions to be designed were assigned as residues that either gained or lost contacts with neighboring residues following helical fusion.

The sequences of individual chains for these designed complexes were submitted to AlphaFold2 for monomeric structure prediction^33^. Complexes where the designed models for each constituent chain possessed low RMSD when aligned to AlphaFold2’s structure predictions (prioritizing alignment to predicted models with high predicted local distance difference test (pLDDT) scores) were selected to order.

### Gene preparation

Genes were codon optimized for bacterial expression and ordered in pET-29b^+^ vector between the NdeI and XhoI restriction sites, with a T7 promoter and kanamycin resistance gene. DHT02, 03, 04 and 05 had an additional de novo minimal protein (design EHEE_rd2_0005)^38^ added to the N terminal of the first chain to increase the molecular weight for SDS-PAGE differentiation. Constructs for co-expression were ordered using ribosome binding sites TAAGAAGGAGATATCATCATG and/or TAAAGAAGGAGATATCATATG in between the chains. The last chain in base and arm sequences had a cleavable N-terminal 6xHis-tag, with recognition sequences for tobacco etch virus or thrombin cleavage. A stop codon was added after the last chain. The ring designs were ordered in the same manner, except here the C-terminal His-tag was kept in frame to reduce the overall DNA synthesis length. An additional Strep-tag II (WSHPQFEK) to allow for Strep-Tactin pull-down was added to the N terminal of chain B for DHT01 arms and DHT01 variant if needed, but was not necessary. Chains for individual expression had either an N-terminal or C-terminal 6xHis-tag. Genes were ordered from Integrated DNA Technologies or GenScript through their custom gene synthesis services. Amino acid sequences for gene inserts and co-expression setups are provided in Supplementary Table 2 (tabs 3 and 4, respectively).

### Protein expression and purification

Plasmids were transformed into either BL21(DE3) or Lemo21(DE3) *E. coli* cells using a 30-s heat shock protocol, added to autoinduction media^39^ and incubated at 225 r.p.m. for 20–22 h at 37 °C. Cell pellets were obtained by centrifugation at 4,000*g* for 15 min, resuspended in 30 ml lysis buffer (25 mM Tris-HCl (pH 8.0), 300 mM NaCl and 20 mM imidazole with added phenylmethylsulfonyl fluoride protease inhibitor), lysed with a sonicator at 85% amplitude with 15 s on/off cycles for a total of 2.5 min (Qsonica) and then spun in the centrifuge at 24,000*g* for 30 min. Cleared lysate was poured over 1–2 ml Ni-NTA resin (QIAGEN) in a column pre-equilibrated with three column volumes of lysis buffer (25 mM Tris-HCl (pH 8.0), 300 mM NaCl and 20 mM imidazole), washed with wash buffer (25 mM Tris-HCl (pH 8.0), 300 mM NaCl and 30 mM imidazole) at 2 × 10 column volumes and eluted with elution buffer (25 mM Tris-HCl (pH 8.0), 300 mM NaCl and 250 mM imidazole) at six column volumes. For Strep-tag purification, Strep-Tactin XT Superflow High Capacity Resin (IBA Lifesciences) was equilibrated with two column volumes of Buffer W (100 mM Tris-HCl (pH 8.0), 150 mM NaCl and 1 mM ethylenediaminetetraacetic acid), IMAC eluate was poured over, the column was washed with five column volumes of Buffer W and protein was eluted with three column volumes of Buffer BXT (100 mM Tris-HCl (pH 8.0), 150 mM NaCl, 1 mM ethylenediaminetetraacetic acid and 50 mM biotin). For tobacco etch virus or thrombin cleavage, imidazole was cleared out through a buffer exchange into TBS buffer (25 mM Tris-HCl (pH 8) and 150 mM NaCl) and enzyme was applied for overnight cleavage. Phenylmethylsulfonyl fluoride was used to stop thrombin cleavage and a second IMAC pull-down was carried out for either cleavage reaction. Flow-through was collected and run through SEC.

### SDS-PAGE

Protein samples were mixed with 2x Laemmli Sample Buffer, heated for 10 min at 95 °C and loaded onto Tris-Glycine gels along with 5 μl Bio-Rad’s Precision Plus Protein Dual Xtra Protein Standards. The gel was run for 30 min at 200 V (Tris-Glycine) and then stained with GenScript’s eStain.

### Reconstitution via annealing

Separately expressed and individually purified components of the heterotrimer can be mixed at a 1:1:1 ratio in a PCR tube and incubated in a thermocycler. The mixture undergoes ~30 min of heating at 90 °C, followed by a gradual cooling by a 2 °C drop every 30 s until 12 °C is reached (resulting in a total of 20 min). For DHT03_2arm_A21/B21/C and subsequent three-arm heterotrimers, 100 µM of each chain was mixed together for reconstitution, while ~30 µM of each chain was mixed together for the DHT03 cyclic rings.

### SEC

An ÄKTA PURE fast protein liquid chromatography system was used. Heterotrimer bases, arm extensions, coiled coil C2 rings and all constructs mentioned in the Supplementary Information were passed through a Cytiva Superdex 200 Increase 10/300 GL column, while C3/C4 rings made from DHT03 were passed through a Cytiva Superose 6 Increase 10/300 GL column. The mobile phase was TBS (25 mM Tris-HCl (pH 8.0) and 100 mM NaCl or 25 mM Tris-HCl (pH 8.0) and 150 mM NaCl). Samples ran at a flow rate of 0.75 ml min^−1^ and fractions were collected at 0.5 ml.

### Statistics and reproducibility

DHT01 was purified and run through SEC at least twice, while DHT01-4arm-01 and DHT01-4arm-02 were purified and run through SEC at least five times. All other heterotrimer bases and arms were purified and run through SEC at least twice. All A2B2 tetramers were purified twice. All A3B3C3 and A4B4C4 rings were purified once in their standard form and again with their DHR-extended versions of chain C. For nsEM class averaging, the total numbers of micrographs collected for each sample are specified in Supplementary Fig. 4.

### Mass spectrometry

The fraction corresponding to the SEC peak was concentrated to 1–2 mg ml^−1^ and run through an Agilent 6230 time-of-flight LC/MS system through an AdvanceBio Desalting-RP column. The mass of the proteins was determined using intact mass spectrometry in positive mode.

### Native mass spectrometry

Samples were analyzed by online buffer exchange nMS^40,41^ to evaluate sample purity and accurately determine oligomeric states^42^. Multiple instruments were used as the analyses were carried out over the duration of the protein design process. The mass spectrometers used for detection were a Q Exactive UHMR system modified with a surface-induced dissociation device and an Exactive Plus EMR system modified with a selection quadrupole and a surface-induced dissociation device (Thermo Fisher Scientific)^42^. The liquid chromatography systems used for the buffer exchange included a Vanquish Duo UHPLC system and a Dionex Ultimate 3000 HPLC system (Thermo Fisher Scientific). A heated electrospray ionization source (HESI-II; Thermo Fisher Scientific) with a spray voltage of ~4 kV was used for ionization. Protein samples stored in Tris buffer were injected (0.1–2.0 µg) onto the liquid chromatography system and exchanged at a flow rate of 100–200 µl min^−1^ into 200 mM ammonium acetate (mobile phase) before ionization. The buffer exchange columns used included self-packed columns with P6 polyacrylamide gel (Bio-Rad) and prototype buffer exchange columns provided by Thermo Fisher Scientific. Instrument parameters were optimized to allow for ion transmission while minimizing unintentional ion activation. Higher-energy collisional dissociation and source fragmentation voltages were used for de-adducting to allow for accurate mass determination. Frequently, collisional dissociation leading to non-covalent fragmentation was used to further validate oligomeric composition. Mass spectra were deconvolved and oligomeric assignments were made using UniDec version 5 and earlier versions (ref. ^43^).

### Circular dichroism

Samples were run over SEC through phosphate-buffered saline (pH 7.4) buffer, concentrated to 0.25 mg ml^−1^ and placed in a 1 mm pathlength cuvette. A JASCO-1500 was used for wavelength scans (190–260 nm) at 25, 75 and 95 °C and a final 25 °C. Temperature melts from 25–95 °C were monitored at 222 nm.

### SAXS

Purified samples were run through 25 mM Tris, 150 mM NaCl and 2% glycerol buffer for SEC^44^. Samples were concentrated using a 10 K molecular weight cutoff benchtop spin concentrator and flow-through from the concentrator was used as a buffer blank. A 1.5–2.5 mg ml^−1^ low concentration range and a 3–6 mg ml^−1^ high concentration range were used for shipping to the SIBYLS MailinSAXS Advanced Light Source in Berkeley, California. The X-ray wavelength (*λ*) was 1.27 Å and the sample-to-detector distance was 1.5 m, corresponding to a scattering vector **q** (**q** = 4π sin *θ*/*λ*, where 2*θ* is the scattering angle) range of 0.01–0.30 Å^−1^. A series of exposures were taken of each well, in equal sub-second time slices: 0.3-s exposures for 10 s, resulting in 32 frames per sample. Collected data were processed using the SIBYLS SAXS FrameSlice server and analyzed using ScÅtter3 (https://bl1231.als.lbl.gov/scatter/). The scattering output was fit to the theoretical design model using the FoXS server (https://modbase.compbio.ucsf.edu/foxs/)^45,46^.

### X-ray crystallography preparation, data collection and analysis

#### Crystallization and structure determination for DHT03

Purified DHT03 protein at a concentration of 40 mg ml^−1^ was used to conduct sitting drop, vapor-diffusion crystallization trials using the JCSG Core I–IV screens (NeXtal Biotechnologies). Crystals of DHT03 grew from drops consisting of 100 nl protein plus 100 nl of a reservoir solution consisting of 0.1 M HEPES (pH 7.5) and 20% (wt/vol) PEG 8000 at 4 °C and were cryoprotected by supplementing the reservoir solution with 15% ethylene glycol. Native diffraction data were collected at Advanced Photon Source (APS) beamline APS-23-ID-B, at wavelength 1.033167 Å, indexed to *P*1 and reduced using the software package XDS^47^ (Table 1). The structure was phased by molecular replacement using Phaser^48^. The core of DHT03_2arm_A21/B21/C (long) was used as a search model. The best solution, with a translation function *Z* score score of 5.8 in Phaser, was AutoBuild by SHELXE and the solution with the best model–map correlation coefficient (0.35) was obtained for Coot^49^ adjustment and refinement using PHENIX^50^.

#### Crystallization and structure determination for DHT03_1arm_A21/B/C

Purified DHT03_1arm_A21/B/C protein at a concentration of 30 mg ml^−1^ was used to conduct sitting drop, vapor-diffusion crystallization trials using the JCSG Core I–IV screens (NeXtal Biotechnologies). Crystals of DHT03_1arm_A21/B/C grew from drops consisting of 100 nl protein plus 100 nl of a reservoir solution consisting of 1 M LiCl, 0.1 M sodium citrate (pH 5) and 20% (wt/vol) PEG 6000 at 4 °C and were cryoprotected by supplementing the reservoir solution with 15% ethylene glycol. Native diffraction data were collected at APS beamline APS-23-ID-B, at wavelength 1.033167 Å, indexed to *P*1 and reduced using XDS^47^ (Table 1). The structure was phased by molecular replacement using Phaser^48^. The core region of a set of ~50 lowest energy predicted models from Rosetta were used as search models. The arm region was subsequently fitted to the density using rigid-body motions in Coot^49^ and refined using PHENIX^50^. The following model building and refinement was done using Coot and PHENIX.

#### Crystallization and structure determination for DHT03_2arm_A21/B21/C (long)

Purified DHT03_2arm_A21/B21/C (long) protein at a concentration of 41 mg ml^−1^ was used to conduct sitting drop, vapor-diffusion crystallization trials using the JCSG Core I–IV screens (NeXtal Biotechnologies). Crystals of DHT03_2arm_A21/B21/C (long) grew from drops consisting of 100 nl protein plus 100 nl of a reservoir solution consisting of 2 M (NH_4_)_2_SO_4_ and 0.1 M sodium acetate (pH 4.6) at 18 °C and were cryoprotected by supplementing the reservoir solution with 2.2 M sodium malonate (pH 5). Native diffraction data were collected at APS beamline APS-23-ID-B, at wavelength 1.033167 Å, indexed to *P*1 and reduced using XDS^47^ (Table 1). The structure was phased by molecular replacement using Phaser^48^. The A chains of a set of ~49 lowest energy predicted models from Rosetta were used as search models. Several of these models gave clear solutions. Chain B and Chain C were fitted manually in Coot^49^ and rigid body refinement was performed with PHENIX^50^. The following model building and refinement was done using Coot and PHENIX.

### Negative stain electron microscopy preparation, data collection and analysis

All SEC-purified samples were diluted to 0.008 mg ml^−1^ in TBS buffer at pH 8.0. For each sample, copper grids (Lacey Carbon, with a 1 µm hole diameter and 5 µm hole spacing) were glow discharged, then 6 μl of diluted sample was applied to each grid and left on for 8 s, then dried with blotter paper. Three rounds of grid staining with uranyl formate (6 μl; 2 mg ml^−1^) were applied to each grid and the grids were left to sit for 8 s before blotting. The grids were left to dry for 5 min.

Data acquisition for nsEM was performed on an FEI Talos L120C transmission electron microscope (120 keV accelerating voltage and 2.7 mm spherical aberration) at a magnification of 92,000× and a pixel size of 1.54 A × 1.54 A. Data collection for selected samples was performed using Thermo Fisher Scientific EPU software. Micrographs were stored as mrc files for subsequent processing.

To process and analyze the data, the collected micrographs were processed and analyzed using the CryoSPARC version 3 software suite^51^. 2D class averages and 3D electron density maps were produced using the pipeline illustrated in Supplementary Fig. 7.

### Reporting summary

Further information on research design is available in the Nature Portfolio Reporting Summary linked to this article.

## Online content

Any methods, additional references, Nature Portfolio reporting summaries, source data, extended data, supplementary information, acknowledgements, peer review information; details of author contributions and competing interests; and statements of data and code availability are available at 10.1038/s41594-022-00879-4.

## Supplementary information

Supplementary Information Supplementary Figs. 1–7, Table 1 and Notes 1 and 2.

Reporting Summary.

Supplementary Table 2 AlphaFold-Multimer predictions for heterotrimer bases (tab 1), AlphaFold2 monomer predictions for rings (tab 2), design sequences for all of the designed proteins (tab 3) and the co-expression setup for multichain protein complexes (tab 4).

Supplementary Code 1 This compressed file contains all PDB files and scripts used in our design protocols and analyses, as well as the final design model outputs.

## Data Availability

Crystal structures have been deposited in the PDB with accession codes 7UPO (DHT03), 7UPQ (DHT03_1arm_A21/B/C) and 7UPP (DHT03_2arm_A21/B21/C long). All of the data are available in the main text or supplementary materials. Source data are provided with this paper.

## Source data

Source Data Fig. 2 Unmodified gels for the insets in Fig. 2c.

## Code availability

The Rosetta software suite is freely available for all non-commercial users, as well as to commercial users for a fee: https://www.rosettacommons.org/. Additional Rosetta scripts can be found in the Supplementary Information. The full code for the WORMS software can be found on GitHub: https://github.com/willsheffler/worms and Zenodo: 10.5281/zenodo.4323517.
