# Supplementary material for: De novo design of obligate ABC-type heterotrimeric proteins
Source: Nat Struct Mol Biol. 2022 Dec 15;29(12):1266–76. doi: 10.1038/s41594-022-00879-4 (PMC9758053; doi:10.1038/s41594-022-00879-4)
Supplement: Supplementary file 1 — Supplementary Figs. 1–7, Table 1 and Notes 1 and 2. [file 41594_2022_879_MOESM1_ESM.pdf]

nature structural & molecular biology

Article https://doi.org/10.1038/s41594-022-00879-4

De novo design of obligate ABC-type heterotrimeric proteins

In the format provided by the authors and unedited

Supplementary Information

De novo design of obligate ABC-Type heterotrimeric proteins

Sherry Bermeo[1,2,3,10], Andrew Favor[1,2,4,10], Ya-Ting Chang[5,10], Andrew Norris[6,7], Scott E. Boyken[1,2], Yang Hsia[1,2], Hugh K. Haddox[1,2], Chunfu Xu[1,2,8], TJ Brunette[1,2], Vicki H. Wysocki[6,7], Gira Bhabha[5], Damian C. Ekiert[5,9], David Baker[1,2,8*]

[1] Department of Biochemistry, University of Washington, Seattle, WA, USA.
[2] Institute for Protein Design, University of Washington, Seattle, WA, USA.
[3] Biological Physics, Structure, and Design Graduate Program, University of Washington, Seattle, WA, USA.
[4] Molecular Engineering & Sciences Institute, University of Washington, Seattle, WA, USA.
[5] Department of Cell Biology, New York University School of Medicine, New York, NY, USA.
[6] Department of Chemistry and Biochemistry, The Ohio State University, Columbus, OH, USA.
[7] Resource for Native Mass Spectrometry Guided Structural Biology, The Ohio State University, Columbus, OH, USA.
[8] Howard Hughes Medical Institute, University of Washington, Seattle, WA, USA.
[9] Department of Microbiology, New York University School of Medicine, New York, NY, USA.
[10] These authors contributed equally.
*email: dabaker@uw.edu

Contents:

- 7 Supplementary Figures
- Supplementary Tables 1-2
- Supplementary Notes 1-2

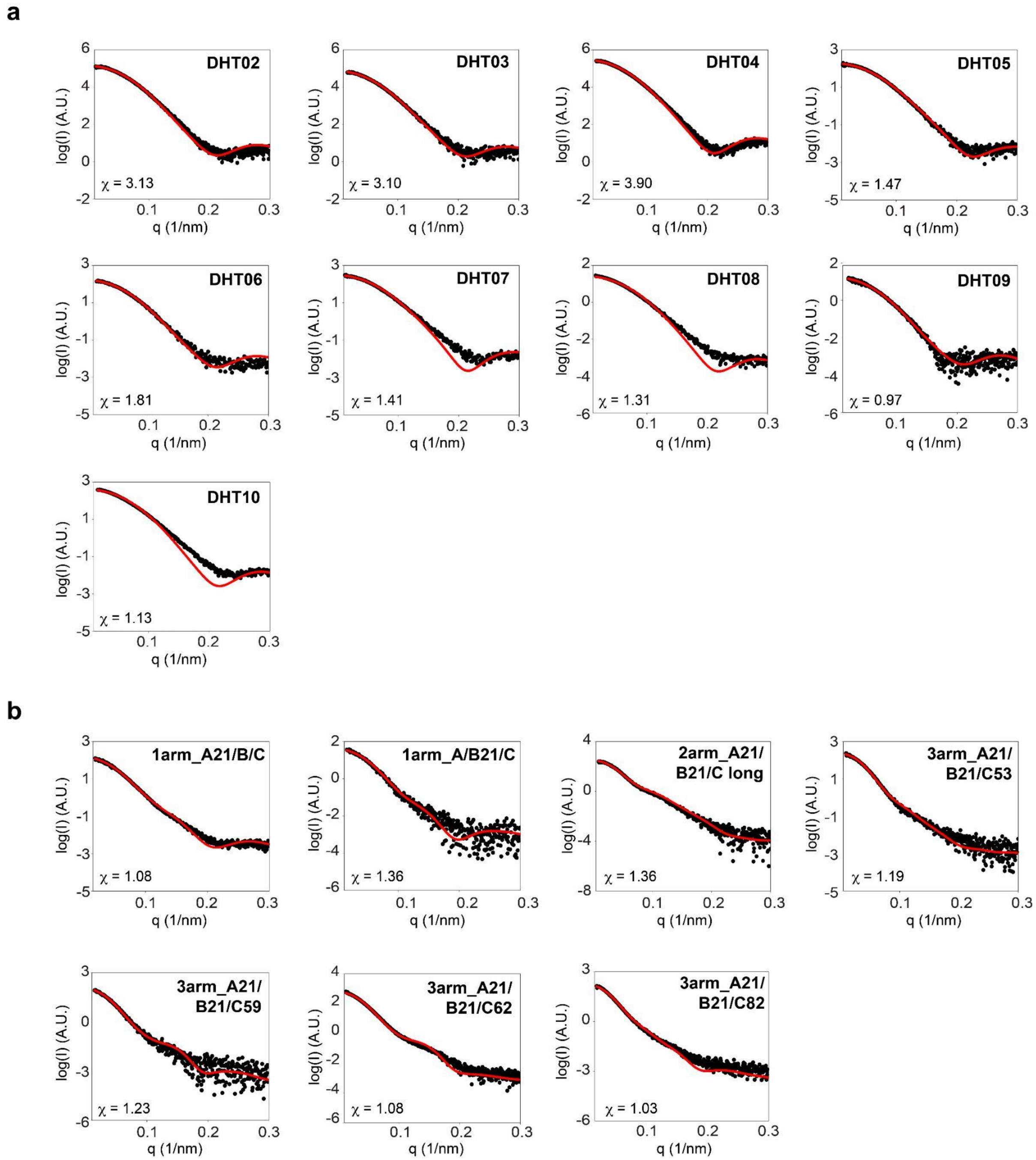

Supplementary Fig. 1 SAXS profiles for heterotrimer designs presented in Figure 3. a Small angle X-ray scattering (SAXS) curve fits for heterotrimer bases indicate good agreement between the design model and experimental data using the FoXS server[1,2], with black dots representing the experimental data and each red line, respectively, calculated from the design model. **b** SAXS profiles for heterotrimer arms built off of DHT03 also indicate overall good

agreement between the design model and the experimental data collected, with further analysis for all designs in Supplementary Table 1.

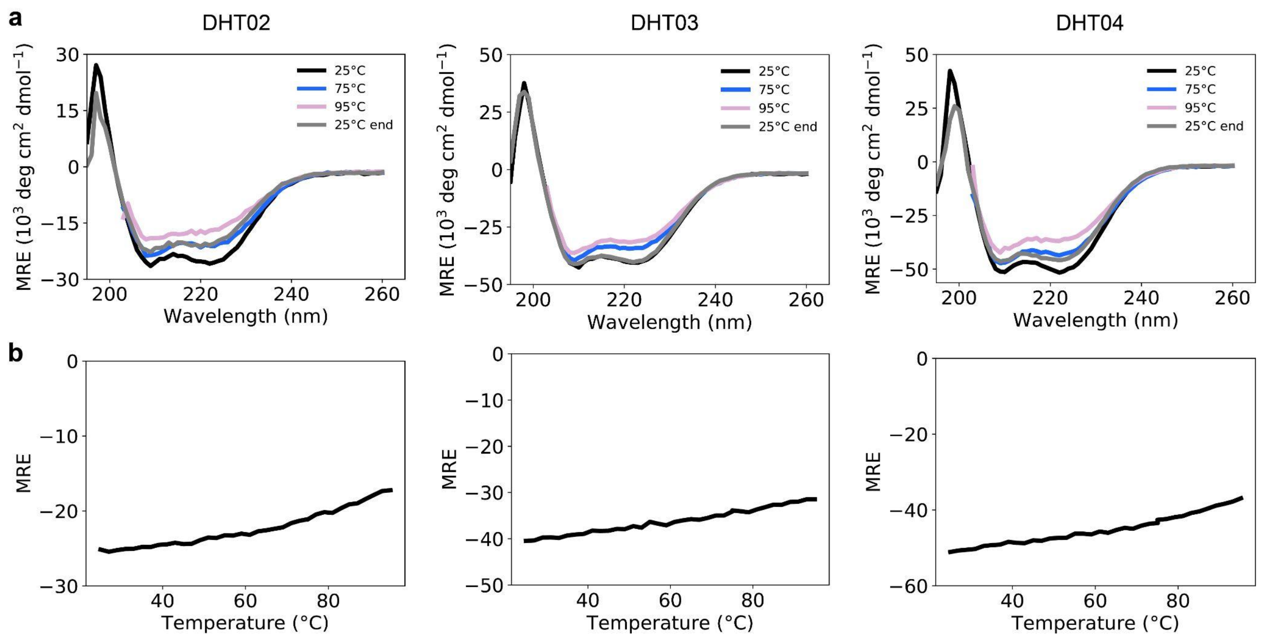

Supplementary Fig. 2 CD plots for three helical bundle heterotrimers. a CD spectra for three helical hairpin heterotrimers at 25°C (black), 75°C (blue), 95°C (pink), and 25°C after cooling (gray). **b** Thermal melting curves measured at 222 nm indicate that all three heterotrimers are thermostable and do not completely unfold at 95°C.

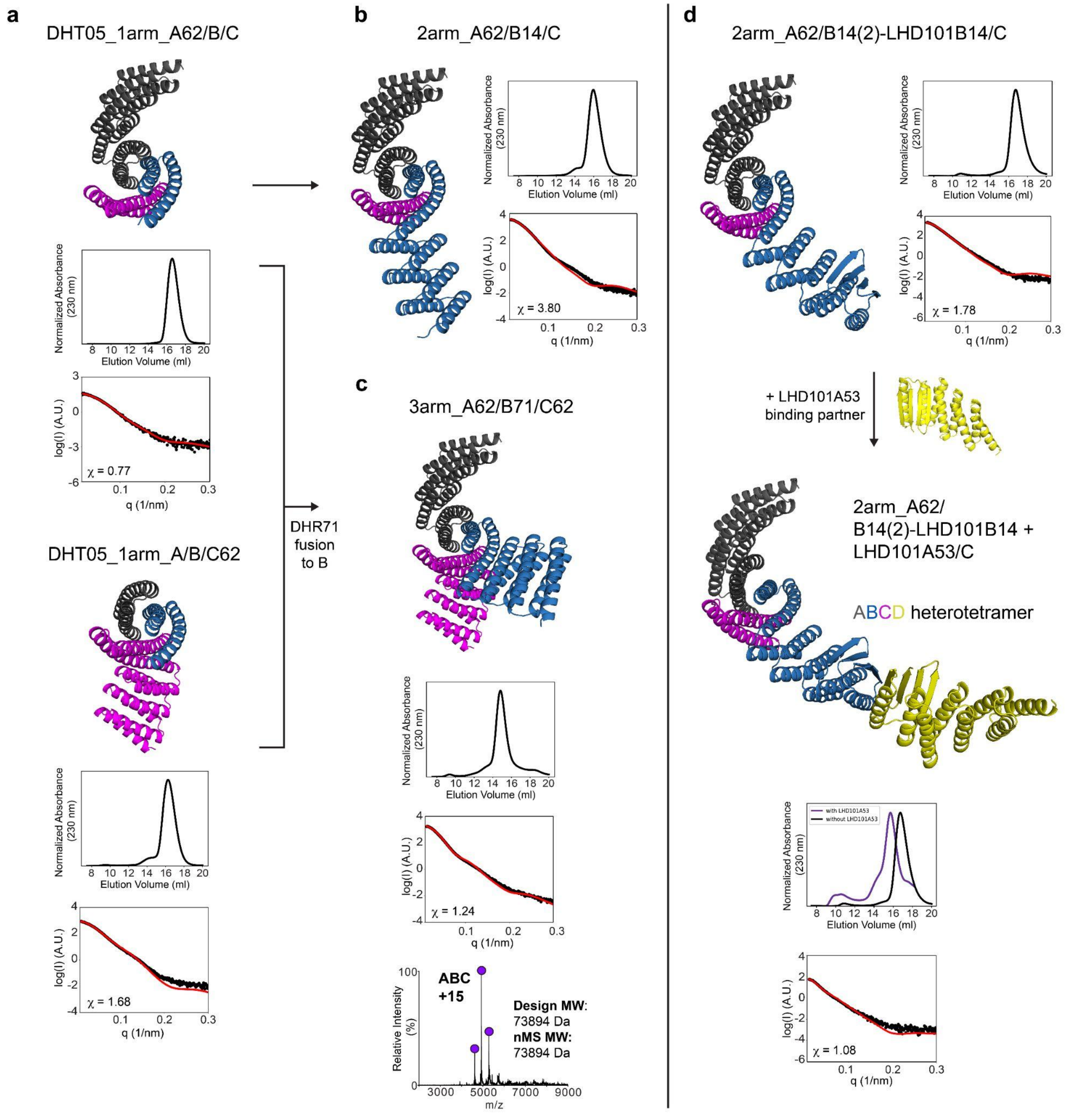

Supplementary Fig. 3 Hierarchical building with DHT05 heterotrimer base. a Hierarchical building approach of DHT05 heterotrimer base is shown, with two successful 1-arm fusions, as determined by monodisperse SEC peaks and SAXS (black dots, experimental data; red line calculated from design model). **b** The one arm construct with a rigid fusion to chain A can be built into a 2-arm construct with another rigid fusion to chain B, as determined by SEC and SAXS. **c** The two 1-arm constructs from **a** can be combined and sustain another rigid fusion to

chain B to create a 3-arm construct, as determined by SEC, SAXS, and native mass spec indicating only the formation of an ABC heterotrimer. **d** A 2-arm construct can be aligned to half of an LHD heterodimer fusion (LHD101B14)[3] with compatible DHR and termini, and then cut and stitched together. This results in an extended 2-arm heterotrimer with one arm capable of binding the respective LHD heterodimer fusion partner, as determined by a leftward shift in the main SEC peak when LHD101A53 is mixed with the co-expressed 2-arm construct. This results in a four component ABCD heterotetramer. Determined radius of gyration via SAXS (Supplementary Table 1) also matches the heterotetramer better compared to that of the heterotrimer above.

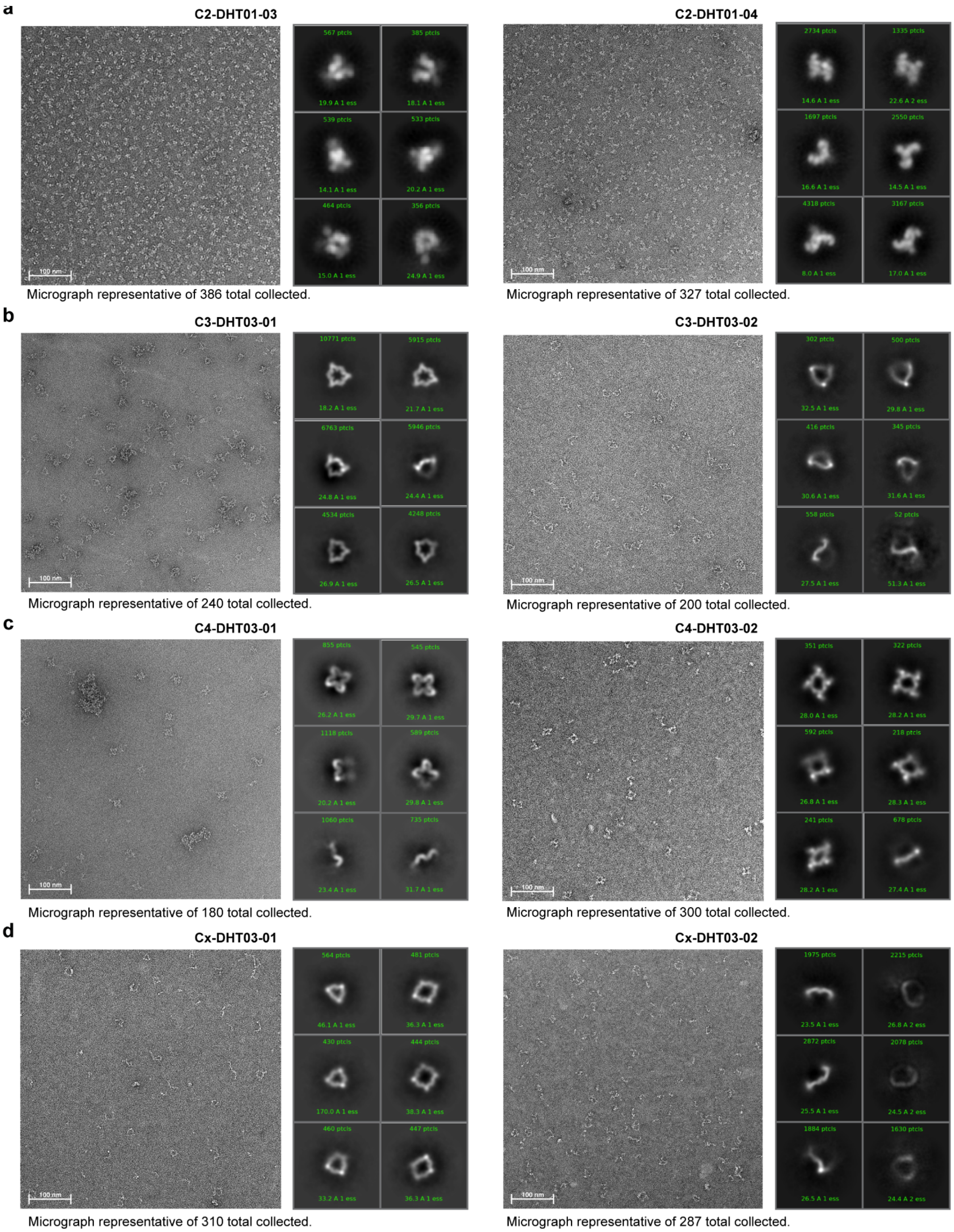

Supplementary Fig. 4 Raw micrographs and 2d class averages. **a** Type-I (left) and type-II (right) A2B2 tetramers, made from heterotrimer DHT01. **b** C3-symmetric A3B3C3 ring

proteins made from heterotrimer DHT03, C3-DHT03-01 (left) and C3-DHT03-02 (right). The helical repeat motifs in C3-DHT03-02 have shorter helices (15 amino acids per helix on average) than in C3-DHT03-01 (20.5 amino acids/helix on average), which likely gives rise to the high flexibility seen in C3-DHT03-02's rings. **c** C4-symmetric A4B4C4 ring proteins made from heterotrimer DHT03, C4-DHT03-01 (left) and C4-DHT03-02 (righ). **d** Ring proteins with mixed oligomeric states, denoted as Cx-DHT03-01 (left) and Cx-DHT03-02 (right), named based on the variation in the observed symmetry.

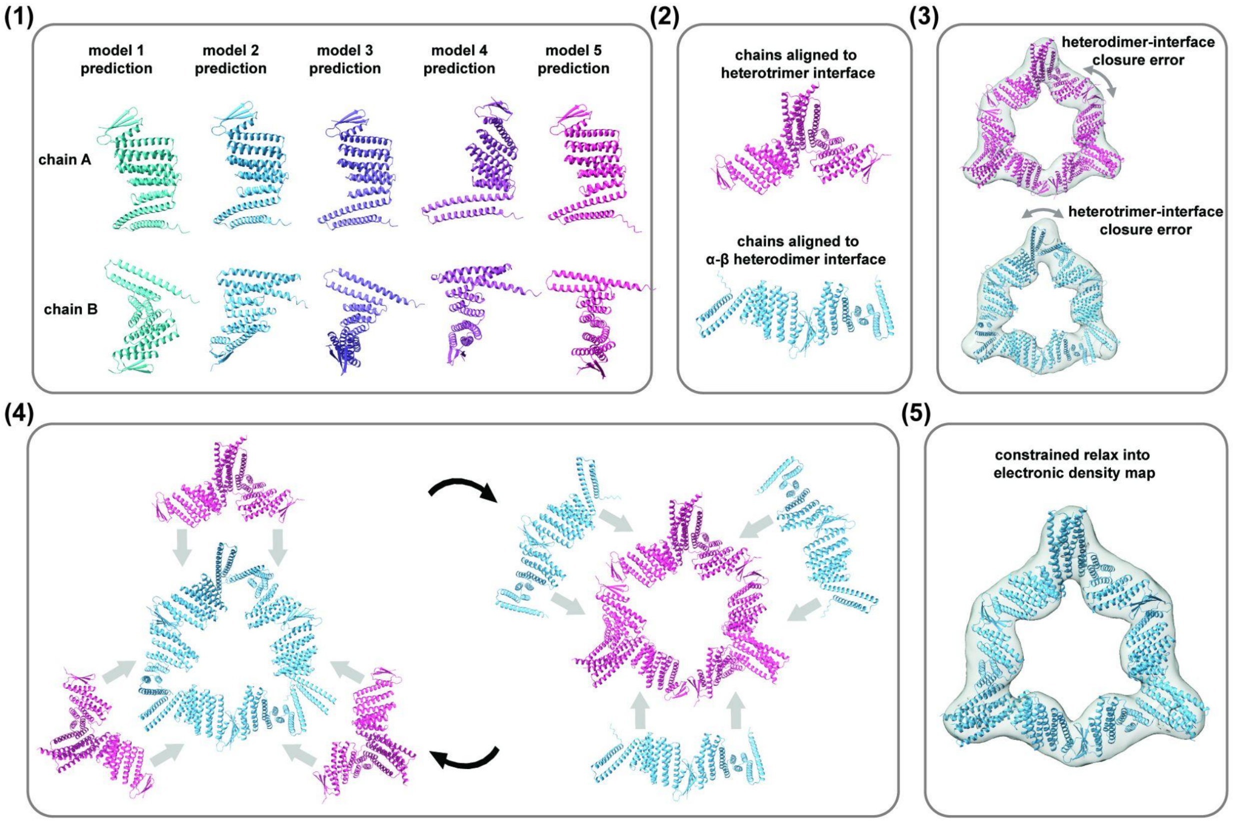

Supplementary Fig. 5 Computational protocol for realignment of PDB models to experimentally observed symmetry states. Several ring-proteins assembled with lower degrees of cyclic symmetry than their design models, as shown my nsEM; C3-DHT03-01 had originally been designed as a C4 ring, while C4-DHT03-01 and C4-DHT03-02 had been designed as C5 rings. Symmetry-correction in the resolved structures followed a 5-step protocol: (1) AlphaFold2[4] was run for each chain (chains A and B) in the ring structure that had undergone WORMS fusion (Supplementary Table 3, tab 2). For each chain, five candidate pdbs were generated, corresponding to predictions performed by AlphaFold2's five different sets of model parameters. (2) Predicted chain structures with high pLDDT were selected, two types of interface-aligned models were created by either aligning the predicted chain models to the DHT03 heterotrimer interface (top) or the LHD heterodimer interface (bottom). (3) Several duplicate copies (one copy per degree of symmetry) of the interface-aligned models containing

both chains A and B were fit to the reconstructed 3D electronic density maps in ChimeraX[5] in order to find reasonable starting positions for further refinement. For the DHT-aligned models, gaps were present at the LHD interface, and for the LHD-aligned models, gaps were present at the DHT interface. (4) Iterative cycles of alignment of between DHT-aligned chains to their LHD-aligned counterparts were performed, which gradually reduced the ring-closure error at both interfaces. (5) In order to refine the structure to satisfy energetics of proper interface formation, and to further optimize the structure's fit within the 3D reconstructed map, Rosetta relax was performed with an additional constraint[6].

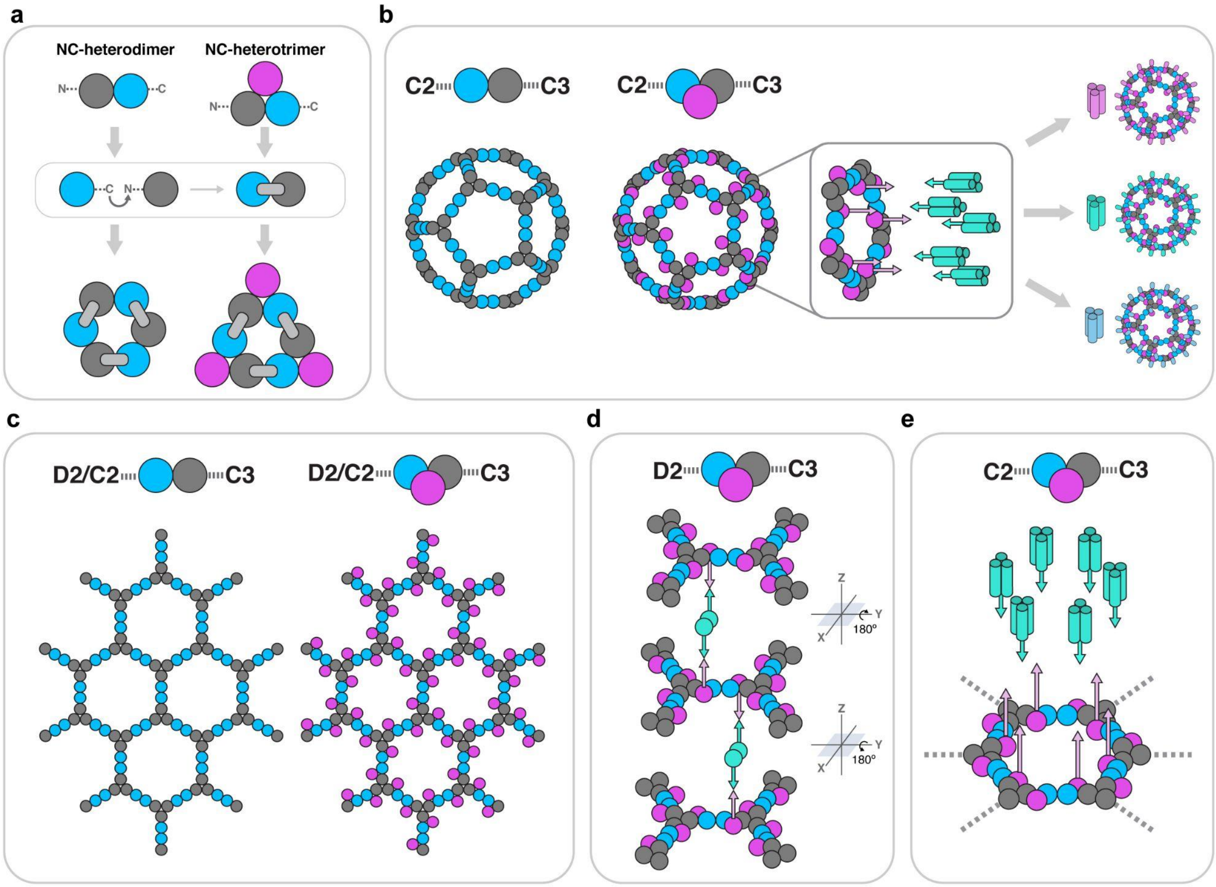

Supplementary Fig. 6 Heterotrimers enable modular functionalization of higher order assemblies. **a** Ring proteins. When using heterodimers to create multimeric complexes with cyclic symmetry, the fusion-accessible termini from each subunit are both used up to form the splice junction. Using a hetero*trimeric* complex to form the interface between ring subunits uses only two chains for ring-closure, leaving the third chain available for fusion to additional scaffolds. **b** Nanocages. Homo-oligomers with cyclic symmetries can be rigidly fused to heterodimers, to form an interface connecting the symmetric centers in protein nanocages, while keeping the point-group interfaces on separate chains within two-component assemblies. However, in these constructs, all fusion-accessible termini are lost by junction formation, which limits the ability to affix further functional motifs or antigens to these structures. In contrast, when using two chains from a hetero*trimer* to form the interface between the chains forming

symmetric interfaces, the third chain remains available for splicing. The fusion-accessibility of the third chain provides extensive design opportunities; for a given nanocage containing a heterotrimeric interface, the third chain can be fused to any number of motifs or antigen-presenting domains, providing many functional opportunities for cage customization without redesigning the base cage scaffold itself. **c** Unbounded assemblies. Two-dimensional lattices can be formed by bridging symmetric point-group interfaces. In these designs, protein chains that form cyclic-symmetric interfaces are connected to chains that form either C2 or D2 symmetric interfaces; interfaces connecting the symmetric centers can be formed by fusion to the chains of a hetero-oligomer. When using heterodimeric interfaces to connect the two types of symmetry-forming chains, a 2-component sheet can be constructed; fusion-accessible termini are used up during splicing, leaving limited opportunities to array functional motifs upon these lattices. When using two chains from a heterotrimer to join the two symmetry-forming components, the third chain is again left accessible for further design opportunities. **d** In the case of sheets containing dihedral interfaces, the cyclic symmetric centers periodically alternate orientation, flipping about the plane of the sheet. When heterotrimers are incorporated into this system, the third chain is available for fusion to an additional C2-symmetric component, forming interfaces in the direction normal to the sheet. Through this arrangement, lattices joined together by heterotrimers can assemble layered sheets, enabling the design of anisotropic biomaterials. **e** In sheets possessing C2 interfaces, asymmetry between the two sides of the sheet is maintained, providing opportunities for one-directional display of functional motifs or antigens. Through the ability to array functional domains in a controlled, periodic manner on one surface of a 2D-lattice, such designs can be utilized for a wide range of applications within protein-based nanotechnology.

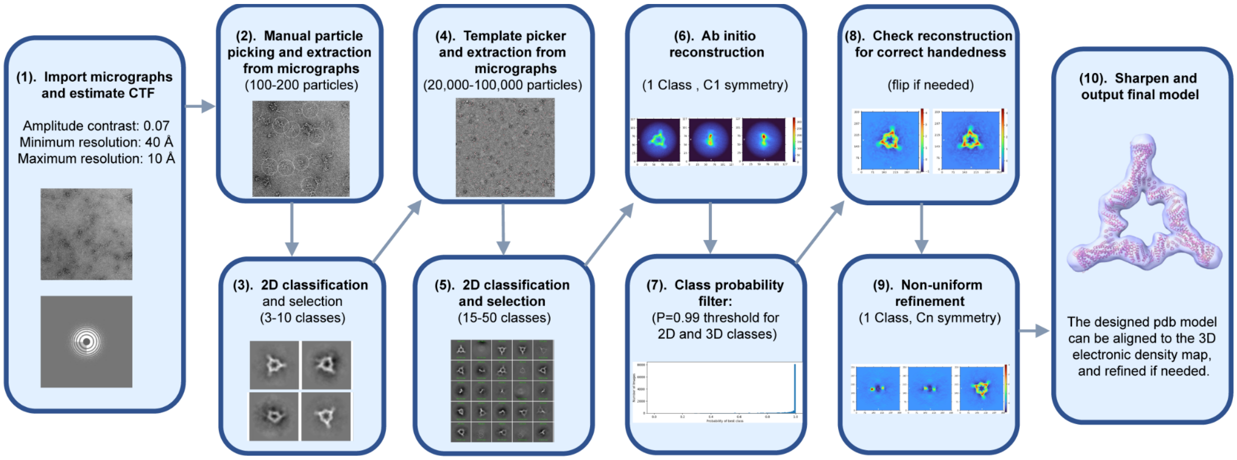

Supplementary Fig. 7 Processing and analysis pipeline for negative stain electron microscopy data. Micrographs were collected on an FEI Talos L120C transmission electron microscope using the Thermo Fisher Scientific EPU software. All steps in the data processing pipeline were performed in CryoSPARC V3[7]. (1) Micrographs were imported as MRC files, and CTF Estimation was performed (0.07 amplitude contrast, 40 Å minimum resolution, 10 Å maximum resolution). (2) A sample of 100-200 particles were manually picked from and extracted from the micrographs with a box size of 256-300 pixels. (3) The selected particles were aligned to generate 10-15 2D class averages, from which 5-10 were selected. (4) The selected classes were used to run CryoSPARC V3's Template Picker algorithm, which led to the extraction 20,000-100,000 particles from the micrographs. (5) 2D class averaging was performed on this larger set of particles, set to generate a maximum of 50 distinct classes, from which 15-25 of the highest quality 2D classes and their constituent particles were selected. (6) Ab initio reconstruction was performed using the particles from the selected classes, without specified symmetry conditions (C1 symmetric processing). (7) Class probability filters were applied with thresholds of P=0.9 for both 2D and 3D class fittings, in order to remove particles that were unlikely to fit the constructed model. (8) The 3D density map was compared to the

design model to check for proper handedness of the reconstruction, and the model was flipped to its mirror symmetry if the reconstruction possessed incorrect chirality. (9) Non-uniform refinement (with symmetry specified) was performed using the selected particles and reconstructed volume, to resolve finer features, and flexible regions. (10) The final model was sharpened, exported, and aligned to the PDB designed model for structural comparison.

Supplementary Table 1. Summary of SAXS analysis

Design name	I(0) (cm-1) [from P(r)]	I(0) (cm-1) [Guinier]	Rg [from P(r)]	Rg [Guinier]	Rg [model]	Rc (Å)	Vr	Porod volume estimate (Å3)	Dmax (Å)	Px
DHT01	19	19.7	28.54	28.85	29.55	11.7	1.4	40166	91	4
DHT01-4arm-01	39.2	47.5	35.51	43.14	43.56	14.8	2.45	186263	94	4
DHT01-4arm-02	27.4	31.8	37.47	44.24	43.67	25.9	2.45	195793	97	3
DHT02	172	172	22.07	21.8	18.75	14.7	1.98	50865	80	4
DHT03	132	129	21.92	21.5	19.28	14.8	1.99	50793	85	3.8
DHT04	232	238	21.56	21.57	18.83	14.9	1.97	50104	76	3.9
DHT05	8.74	9.12	21.02	21.07	18.7	13.9	3.24	54102	65	3.5
DHT06	2.14	2.16	19.66	18.59	18.83	13.8	1.58	40741	61	3.9
DHT07	11.1	11.9	20.91	20.97	18.69	13.4	4.06	47859	69	3.4
DHT08	4.23	4.32	21.26	21.32	18.42	13.2	4.15	43482	73	3.9
DHT09	7.11	6.92	22.46	22.1	18.84	14.6	4.7	56189	79	3.8
DHT10	8.55	9.35	20.7	21.19	18.12	13.8	3.93	45697	67	3.8
DHT03_A21/B/C	8.09	8.61	26.01	26.01	23.93	16.9	3.1	88917	85	3.4
DHT03_A/B21/C	5.07	5.07	27.72	28.95	24.22	18	5.94	99586	85	2.9
DHT03_A21/B21/C long	11.9	11.8	35.76	36.31	37.31	24.2	3.45	131455	97	3.8
DHT03_3arm_A21/B21/C53	2.29	2.49	33.39	35.5	32.41	22.7	3.5	136860	93	2.8
DHT03_3arm_A21/B21/C59	10.2	9.33	34.68	37	33.61	24.2	5.43	118238	96	3.5
DHT03_3arm_A21/B21/C62	14.6	16.1	33.67	36.26	33.48	21.8	4.34	131400	98	2.9
DHT03_3arm_A21/B21/C82	8.56	9.31	35.17	38.76	35.85	24.2	3.57	189747	93	2.6
DHT03_2arm_A21/Bt18/C	23	22	32.67	33.19	30.02	21	2.27	137036	97	3.8
DHT03_2arm_A21/B14/C	59.2	59.3	28.83	29.26	26.17	20.2	2.74	131987	91	3.6
DHT03_2arm_A21/B62/C	60.7	58.4	31.15	31.32	27.6	20.2	2.55	102992	97	3.9
DHT05_1arm_A62/B/C	5.51	5.23	25.2	24.5	23.99	15.9	1.67	64714	93	3.2
DHT05_1arm_A/B/C62	18	19.4	28.22	28.38	28	17.1	2.05	67635	91	3.9
DHT05_2arm_A62/B14/C	35.3	36.4	31.54	32.56	31.03	20.7	3.17	103932	97	4
DHT05_3arm_A62/B71/C62	25.8	27.9	35.75	37.94	34.99	22.3	2.37	108636	97	3.8
DHT05_2arm_A62/B14(2)-LHD101B14/C	28	30.9	32.71	35.53	33.02	18.5	1.92	106756	97	3.9
DHT05_2arm_A62/B14(2)-LHD101B14+LHD101A53/C	4.86	6.84	35.39	47.64	46.9	18.3	4.82	148769	95	2.6
C2-DHT01-01	82.5	99.7	39.9	53	48.51	41.07	5.41	354220	97	4.1
C2-DHT01-02	114	142	44.62	53.5	48.28	41.46	5.86	406074	97	3.8
C2-DHT01-03	129	157	40.85	51.05	44.52	33.7	5.27	360645	97	3.6
C2-DHT01-04	92.1	119	40.81	52.1	48.92	36.84	2.86	373163	97	3.9

Rg = radius of gyration; Rc = cross-sectional radius of gyration determined from Guinier fitting; Px = Porod exponent.

Supplementary Table 2. (provided separately as a multi-tab xlsx file)

Supplementary Note 1. WORMS database file entries for DHT, LHD, and DHR inputs

DHT heterotrimer database contents (DHT_inputs.json):


```
{"file": "DHT01-4arm-02.pdb",
  "class": ["DHT_NC"],
  "type": "DHT_NC",
  "base": "",
  "name": "DHT01-4arm-02",
  "components": [""],
  "validated": true,
  "protocol": "",
  "connections":[{"chain": 1, "direction": "C", "residues": ["-79:"]},
             {"chain": 2, "direction": "N", "residues": [":110"]},
             {"chain": 3, "direction": "N", "residues": [":110"]},
             {"chain": 3, "direction": "C", "residues": ["-79:"]}]},
{"file": "DHT03_2arm_fixed_xtal.pdb",
  "class": ["DHT_NN"],
  "type": "DHT_NN",
  "base": "DHT03_2arm_fixed_xtal",
  "name": "DHT03_2arm_fixed_xtal",
  "components": [""],
  "validated": true,
  "protocol": "",
  "connections":[{"chain": 1, "direction": "N", "residues": [":170"]},
                {"chain": 2, "direction": "N", "residues": [":170"]}]}
```


LHD heterodimer database contents (LHD_inputs.json**):**


```
{"file": "LHD274_A64_B82.pdb",
  "class": ["LHD_CC"],
  "type": "LHD_CC",
  "base": "LHD274_A64_B82",
  "name": "LHD274_A64_B82",
  "components": [""],
  "validated": false,
  "protocol": "",
  "connections":[{"chain": 1, "direction": "C", "residues": ["-187:"]},
                {"chain": 2, "direction": "C", "residues": ["-196:"]}]},
{"file": "LHD29_A53_B53.pdb",
  "class": ["LHD_CC"],
  "type": "LHD_CC",
  "base": "LHD29_A53_B53",
  "name": "LHD29_A53_B53",
  "components": [""],
  "validated": false,
  "protocol": "",
  "connections":[{"chain": 1, "direction": "C", "residues": ["-140:"]},
                {"chain": 2, "direction": "C", "residues": ["-138:"]}]},
{"file": "LHD274_A76_B82.pdb",
  "class": ["LHD_CC"],
  "type": "LHD_CC",
  "base": "LHD274_A76_B82",
  "name": "LHD274_A76_B82",
```

```
    "components": [""],
    "validated": false,
    "protocol": "",
    "connections":[{"chain": 1, "direction": "C", "residues": ["-185:"]},
                   {"chain": 2, "direction": "C", "residues": ["-196:"]}]}
```

DHR monomer database contents (DHR_inputs.json**):**

```
  {"file": "DHR64_trim.pdb",
    "name": "DHR64",
    "class": ["Monomer"],
    "type": "repeat_protein" ,
    "base": "" ,
    "components": ["DHR64"],
    "validated": true,
    "protocol": "n/a",
    "connections": [
     {"chain": 1, "direction": "N", "residues":[":168"]},
     {"chain": 1, "direction": "C", "residues":["-168:"]}]},
  {"file": "DHR70_trim.pdb",
    "name": "DHR70",
    "class": ["Monomer"],
    "type": "repeat_protein" ,
    "base": "" ,
    "components": ["DHR70"],
    "validated": true,
    "protocol": "n/a",
    "connections": [
     {"chain": 1, "direction": "N", "residues":[":147"]},
     {"chain": 1, "direction": "C", "residues":["-147:"]}]},
  {"file": "DHR71_trim.pdb",
    "name": "DHR71",
    "class": ["Monomer"],
    "type": "repeat_protein" ,
    "base": "" ,
    "components": ["DHR71"],
    "validated": true,
    "protocol": "n/a",
    "connections": [
     {"chain": 1, "direction": "N", "residues":[":147"]},
     {"chain": 1, "direction": "C", "residues":["-147:"]}]}
```

Supplementary Note 2. WORMS flag file specifications for different cyclic searches

C2-rings (A2B2) using coiled-coil heterotrimer:

```
--geometry Cyclic(2)
--bbconn
       _C DHT_NC
       NC Monomer
       N_ DHT_NC
--monte_carlo 0
--tolerance 1.5
--splice_ncontact_cut 10
--splice_ncontact_no_helix_cut 4
--splice_nhelix_contacted_cut 2
--postfilt_splice_ncontact_cut 10
--postfilt_splice_ncontact_no_helix_cut 4
--postfilt_splice_nhelix_contacted_cut 2
--dbfiles
       DHT_inputs.json
       DHR_inputs.json
```

C3-rings (A3B3C3) using helical bundle heterotrimer and α-β heterodimer

```
--geometry Cyclic(3)
--bbconn
       _N DHT_NN
       CC LHD_CC
       N_ DHT_NN
--monte_carlo 0
--tolerance 1.5
--splice_ncontact_cut 10
--splice_ncontact_no_helix_cut 4
--splice_nhelix_contacted_cut 2
--postfilt_splice_ncontact_cut 10
--postfilt_splice_ncontact_no_helix_cut 4
--postfilt_splice_nhelix_contacted_cut 2
--dbfiles
       DHT_inputs.json
       LHD_inputs.json
```

C4-rings (A4B4C4) using helical bundle heterotrimer and α-β heterodimer

```
--geometry Cyclic(4)
--bbconn
       _N DHT_NN
       CC LHD_CC
       N_ DHT_NN
--monte_carlo 0
--tolerance 1.5
--splice_ncontact_cut 10
--splice_ncontact_no_helix_cut 4
--splice_nhelix_contacted_cut 2
--postfilt_splice_ncontact_cut 10
--postfilt_splice_ncontact_no_helix_cut 4
--postfilt_splice_nhelix_contacted_cut 2
--dbfiles
       DHT_inputs.json
       LHD_inputs.json
```

Supplementary Code:

Information for downloading pdb files and design scripts: all pdb files and scripts used in our design protocols are provided in the Supplementary Code zip-file.

Subdirectory organization:

- pdbs/
  - arm_pdbs/
  - base_pdbs/
  - crown_pdbs/
  - DHT03_partitions_pdbs/
  - AF-Multimer for base_pdbs/
  - AF-Monomer for worms fusions/
- scripts/
  - for_arms/
  - for_bases/
  - for_rings/

References:

1. Schneidman-Duhovny, D., Hammel, M., Tainer, J.A., and Sali, A.. Accurate SAXS profile computation and its assessment by contrast variation experiments. *Biophysical Journal* **105**(4), 962-974 (2013).

2. Schneidman-Duhovny D., Hammel, M., Tainer, J.A., and Sali, A. FoXS, FoXSDock and MultiFoXS: Single-state and multi-state structural modeling of proteins and their complexes based on SAXS profiles. *NAR* **44**(1), W424-W429 (2016).

3. Sahtoe, D.D. & Praetorius, F. et al. Reconfigurable asymmetric protein assemblies through implicit negative design. *Science* **375**(6578), eabj7662 (2022).

4. Jumper, J. et al. Highly accurate protein structure prediction with AlphaFold. *Nature* **596**, 583-589 (2021).

5. Pettersen, E.F. et al. UCSF ChimeraX: Structure visualization for researchers, educators, and developers. *Prot. Sci*. **30**(1), 70-8 (2021).

6. DiMaio, F., Tyka, M.D., Baker, M.L., Chiu, W, Baker, D. Refinement of protein structures into low-resolution density maps using rosetta. *Journal of Molecular Biology* **392**(1), 181-190 (2009).

7. Punjani, A., Rubinstein, J.L., Fleet, D.J., Brubaker, M.A. cryoSPARC: algorithms for rapid unsupervised cryo-EM structure determination. *Nature Methods* **14**(3), 290-296 (2017).
