# Supplementary material for: De novo design of obligate ABC-type heterotrimeric proteins
Source: Nat Struct Mol Biol. 2022 Dec 15;29(12):1266–76. doi: 10.1038/s41594-022-00879-4 (PMC9758053; doi:10.1038/s41594-022-00879-4)
Supplement: Supplementary file 2 — Reporting Summary. [file 41594_2022_879_MOESM2_ESM.pdf]

nature portfolio

Corresponding author(s): David Baker

Last updated by author(s): 10/14/2022

Reporting Summary

Nature Portfolio wishes to improve the reproducibility of the work that we publish. This form provides structure for consistency and transparency in reporting. For further information on Nature Portfolio policies, see our Editorial Policies and the Editorial Policy Checklist.

Statistics

For all statistical analyses, confirm that the following items are present in the figure legend, table legend, main text, or Methods section.

n/a	Confirmed	
☐	☒	The exact sample size (*n*) for each experimental group/condition, given as a discrete number and unit of measurement
☐	☒	A statement on whether measurements were taken from distinct samples or whether the same sample was measured repeatedly
☒	☐	The statistical test(s) used AND whether they are one- or two-sided<br>*Only common tests should be described solely by name; describe more complex techniques in the Methods section.*
☒	☐	A description of all covariates tested
☒	☐	A description of any assumptions or corrections, such as tests of normality and adjustment for multiple comparisons
☒	☐	A full description of the statistical parameters including central tendency (e.g. means) or other basic estimates (e.g. regression coefficient) AND variation (e.g. standard deviation) or associated estimates of uncertainty (e.g. confidence intervals)
☒	☐	For null hypothesis testing, the test statistic (e.g. *F*, *t*, *r*) with confidence intervals, effect sizes, degrees of freedom and *P* value noted<br>*Give P values as exact values whenever suitable.*
☒	☐	For Bayesian analysis, information on the choice of priors and Markov chain Monte Carlo settings
☒	☐	For hierarchical and complex designs, identification of the appropriate level for tests and full reporting of outcomes
☒	☐	Estimates of effect sizes (e.g. Cohen's *d*, Pearson's *r*), indicating how they were calculated

Our web collection on statistics for biologists contains articles on many of the points above.

Software and code

Policy information about availability of computer code

Data collection	Rosetta software (https://www.rosettacommons.org); WORMS software (https://github.com/willsheffler/worms ; https://doi.org/10.5281/zenodo.4323517.)
Data analysis	Python3.8; PyRosetta; Rosetta calculations; UniDec v5; Coot 0.9.4.1; Phenix 1.13; Matplotlib 3.5; Pymol 2.4.1; ScÅtter3; Molprobity 4.02b-467.

For manuscripts utilizing custom algorithms or software that are central to the research but not yet described in published literature, software must be made available to editors and reviewers. We strongly encourage code deposition in a community repository (e.g. GitHub). See the Nature Portfolio guidelines for submitting code & software for further information.

Data

Policy information about availability of data

All manuscripts must include a data availability statement. This statement should provide the following information, where applicable:

- Accession codes, unique identifiers, or web links for publicly available datasets
- A description of any restrictions on data availability
- For clinical datasets or third party data, please ensure that the statement adheres to our policy

Crystal structures have been deposited in the Protein Data Bank with accession codes 7UPO (DHT03), 7UPQ (DHT03_1arm_A21/B/C), and 7UPP (DHT03_2arm_A21/B21/C long). All data are available in the main text or the supplementary materials. Worms database-file contents are provided in Supplementary Note 1, with contents corresponding to the pdb files included in the source data.

March 2021

Field-specific reporting

Please select the one below that is the best fit for your research. If you are not sure, read the appropriate sections before making your selection.

- [x] Life sciences
- [ ] Behavioural & social sciences
- [ ] Ecological, evolutionary & environmental sciences

For a reference copy of the document with all sections, see nature.com/documents/nr-reporting-summary-flat.pdf

Life sciences study design

All studies must disclose on these points even when the disclosure is negative.

Sample size	For heterotrimer bases, 85 designs were ordered ; for A2B2 tetramers, 12 designs were ordered; for 3-component ring proteins, 10 designs were ordered. There was no explicit statistical method applied when choosing the number of designs to order, but Rosetta metrics were used to determine which designs to order in each case. Smaller sets of designs were chosen as an example to highlight a presented idea, with the impression that larger sample sizes would likely lead to similar results.
Data exclusions	There is no data exclusion in this study.
Replication	DHT01 was purified and run through SEC at least twice, while DHT01-4arm-01 and DHT01-4arm-02 were purified and run through SEC and purified at least five times. All other heterotrimer bases and arms were purified and run through SEC at least twice. All A2B2 tetramers were purified twice. All A3B3C3 and A4B4C4 rings were purified once in their standard form, and again with their DHR-extended versions of chain C. Re-expression of proteins described in this study led to the same results as the original batch tested. Conditions were not varied when re-expression was performed. For nsEM data collection, the number of micrographs collected for each single sample is specified in Supplementary Figure 4.
Randomization	Randomization is not relevant to study. This is an observational study, which does not involve population-treatment or evaluation of conditional effects.
Blinding	Blinding is not relevant to study; keeping track of each protein's identity over the course of experimental characterization was necessary for analyzing the structure and biophysical properties of such designs.

Reporting for specific materials, systems and methods

We require information from authors about some types of materials, experimental systems and methods used in many studies. Here, indicate whether each material, system or method listed is relevant to your study. If you are not sure if a list item applies to your research, read the appropriate section before selecting a response.

Materials & experimental systems

n/a	Involved in the study	
☒	☐	Antibodies
☒	☐	Eukaryotic cell lines
☒	☐	Palaeontology and archaeology
☒	☐	Animals and other organisms
☒	☐	Human research participants
☒	☐	Clinical data
☒	☐	Dual use research of concern

Methods

n/a	Involved in the study	
☒	☐	ChIP-seq
☒	☐	Flow cytometry
☒	☐	MRI-based neuroimaging
