# Supplementary material for: De novo design of obligate ABC-type heterotrimeric proteins
Source: Nat Struct Mol Biol. 2022 Dec 15;29(12):1266–76. doi: 10.1038/s41594-022-00879-4 (PMC9758053; doi:10.1038/s41594-022-00879-4)
Supplement: Source Data Fig. 2 — Unmodified gels for the insets in Fig. 2c. [file 41594_2022_879_MOESM5_ESM.pdf]

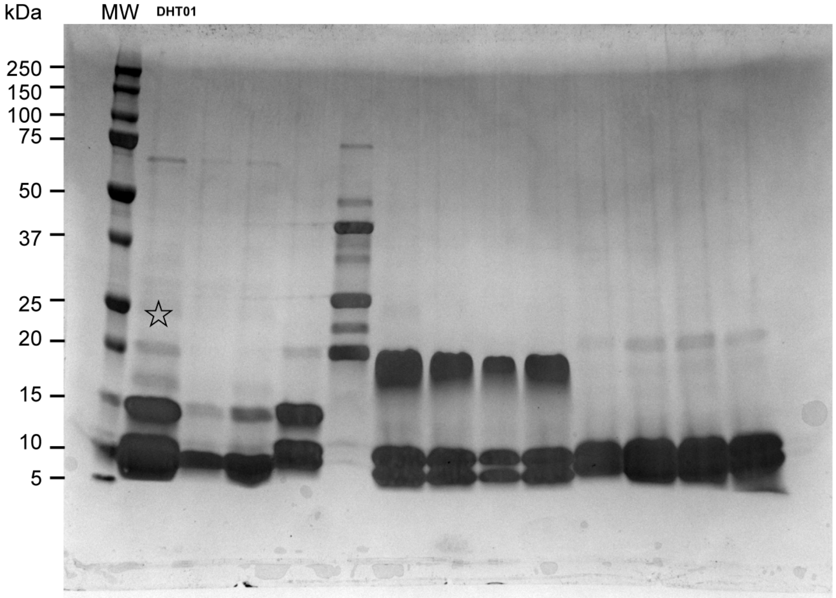

Lane 2 of uncropped gel contains DHT01 protein sample, as seen in Figure 2c inset.

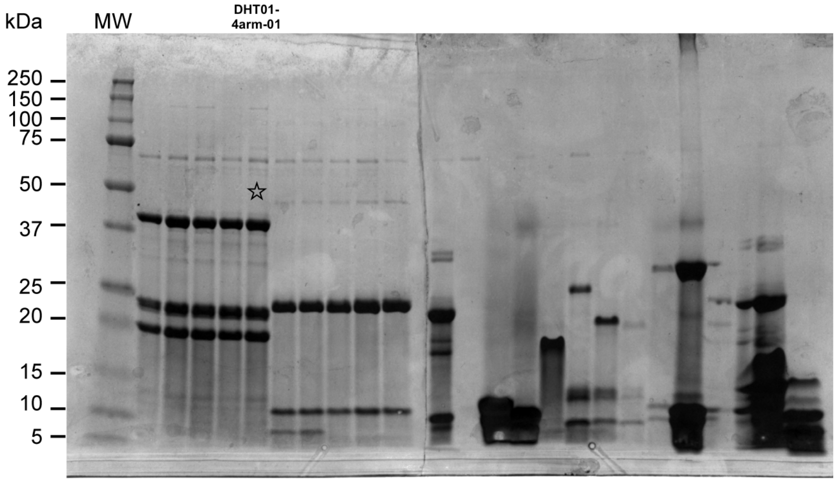

Lane 6 of uncropped gel contains DHT01-4arm-01 protein sample, as seen in Figure 2c inset.

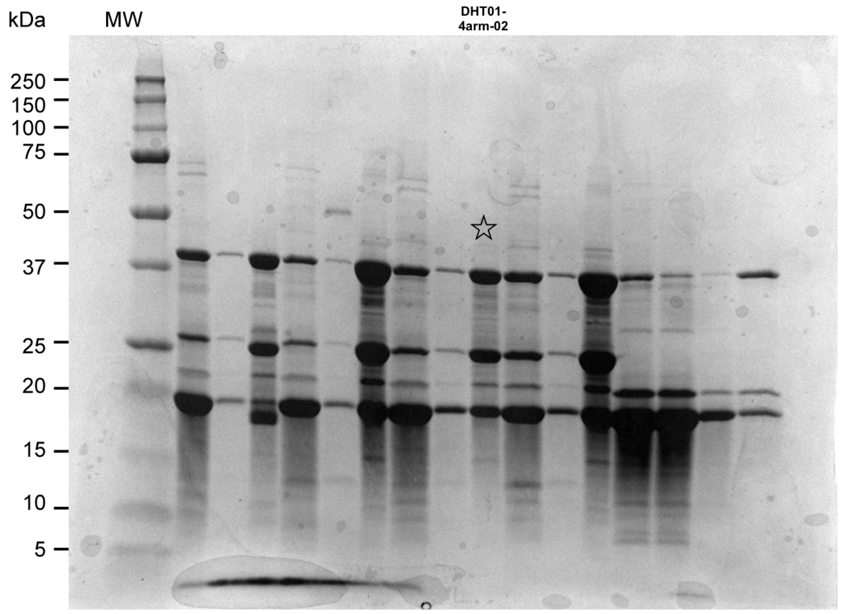

Lane 10 of uncropped gel contains DHT01-4arm-02 protein sample, as seen in Figure 2c inset.
